# The Rising Burden of Motorcycle Crashes and Rider Injuries in Türkiye (2018–2025): An Ecological Analysis of Routinely Collected National Statistics with Implications for Injury Prevention and Trauma Care

**DOI:** 10.3390/healthcare14183072

**Published:** 2026-09-18

**Authors:** Mustafa Doğan, Muhammed Emin Parlak, Yasin Etli

**Affiliations:** 1 Department of Forensic Medicine, Faculty of Medicine, Niğde Ömer Halisdemir University, Niğde 51240, Türkiye; mustafa.dogan@ohu.edu.tr (M.D.); eminparlak@ohu.edu.tr (M.E.P.); 2 Department of Forensic Medicine, Faculty of Medicine, Van Yüzüncü Yıl University, Van 65080, Türkiye

**Keywords:** motorcycles, road traffic accidents, injury prevention, trauma care, vulnerable road users, trauma systems, occupational accidents, couriers, epidemiology, Türkiye

## Abstract

**Highlights:**

**What are the main findings?**

- Rider deaths rose by 62% between 2018 and 2025 (823 to 1334; +93% from the 2019 low) while the fleet more than doubled and fleet-adjusted mortality fell by about 1.7% per year—the rise is exposure-driven.
- Recorded injured riders tripled to over 130,000 per year, the fatality proportion (deaths per 100 casualties) halved, and roughly two-thirds to 71% of rider deaths occurred after arrival at medical care.

**What are the implications of the main findings?**

- Prevention should manage exposure—licensing and delivery-platform regulation—not rider behaviour alone.
- Most remaining mortality lies within the trauma-care pathway, and a five-fold provincial gradient in the fatality proportion shows where post-crash care investment matters most.

**Abstract:**

**Background/Objectives:** Türkiye’s registered motorcycle fleet more than doubled after the COVID-19 pandemic amid rapid growth of the platform delivery economy. In a national ecological analysis of routinely collected official statistics (2018–2025), we quantified the resulting mortality and injury burden; the injury burden is operationalised as police-recorded injured persons, a proxy for emergency care demand rather than measured hospital contacts. **Methods:** Rider deaths and injuries were modelled by Poisson/quasi-Poisson regression with the registered fleet as an offset (primary mortality window 2019–2025; 2018 in sensitivity analyses). Non-motorcycle deaths served as a contemporaneous comparison series, compared formally through a pooled interaction model; the insured fleet served as an alternative exposure denominator; and the care pathway, courier occupational records, province-level correlates (81 provinces), and 2030 projections were also analysed. **Results:** Rider deaths rose from 823 (2018) to 1334 (2025)—from 12.3% to 22.1% of all road deaths—while the fleet grew from 3.2 to 7.1 million. The fleet-adjusted mortality rate declined by about 1.7% per year (2019–2025: incidence rate ratio (IRR) 0.983, 95% confidence interval (CI) 0.971–0.995); its trend did not differ measurably from the comparison series (ratio of trends 1.011, 95% CI 0.951–1.075), and fleet growth alone predicted at least the entire rise in deaths. Injured riders more than tripled (fleet-adjusted IRR 1.071, 95% CI 1.051–1.091, for 2018–2025; 1.080, 1.058–1.103, for 2019–2025), the fatality proportion halved (2.02 to 1.01 deaths per 100 reported casualties), and roughly two-thirds to 71% of deaths in every year occurred after arrival at medical care, with death rates falling in both the scene and post-admission phases. Courier occupational accident rates rose from 13.8 to 19.1 per 1000 insured workers. Provincial fatality proportions varied five-fold, with urbanisation the only robust correlate (rate ratio (RR) 0.85 per standard deviation (SD)). Trend-based planning scenarios project 237,000–344,000 injured riders annually by 2030. **Conclusions:** An exposure-driven mortality surge with a steep morbidity increase is concentrating within the trauma-care pathway; priorities are exposure management, delivery-sector regulation, occupational registration, and post-crash care capacity.

## 1. Introduction

Road traffic injuries remain one of the leading causes of death and disability worldwide, claiming an estimated 1.19 million lives each year according to the WHO Global Status Report on Road Safety 2023 [1], while the Global Burden of Disease 2023 study independently estimates approximately 1.34 million road-injury deaths and 50.9 million incident cases for 2023 [2,3]. Progress has been profoundly uneven across road-user groups: while occupant fatalities have declined in many settings, deaths among riders of powered two-wheelers have risen in both absolute and relative terms, and motorcyclists now constitute one of the largest single groups of road deaths globally: in GBD 2023, motorcyclists accounted for 13.2% of global road-injury incident cases but 23.8% of road-injury deaths [2,4,5]. Against the targets of the United Nations Decade of Action for Road Safety 2021–2030 and Sustainable Development Goal 3.6—a 50% reduction in road deaths by 2030—the motorcycle has become the pivotal obstacle in a growing number of countries [2,6,7].

A major driver of this divergence is motorisation itself. In low- and middle-income countries, rapid growth of the motorcycle fleet has repeatedly been identified as the profile most strongly associated with rising road-death rates [8]. The trajectory is well-documented in Brazil, where explosive fleet growth transformed the composition of traffic mortality within two decades [9,10,11], and similar exposure-driven patterns have been described across Latin America and the Caribbean [12] and in the motorcycle-dependent traffic systems of Southeast Asia [13]. These experiences underline a point of direct policy relevance: when mortality grows in proportion to the fleet, the decisive levers lie in managing exposure and the systems that generate it, whereas a rising per-vehicle risk would instead point towards deteriorating rider behaviour or road conditions. Distinguishing between these two mechanisms therefore has practical consequences for prevention planning.

A second, more recent force is the platform-based delivery economy. Online food and parcel delivery expanded rapidly during and after the COVID-19 pandemic [14], creating a large occupational population of motorcycle riders whose working conditions—time pressure, per-drop payment, incentive and bonus schedules, long hours, and algorithmic management—are increasingly recognised as occupational health hazards [15,16,17]. Emergency department series and cohort studies from Australia, Korea, and Asia consistently show that delivery riders carry a distinct and substantial crash and injury burden [18,19,20], with elevated rates of traffic violations, fatigue-related crashes, and pressure-mediated risk-taking [21,22,23]. Because much of this workforce is precariously employed, its injuries are also systematically under-captured by official occupational-accident registries [24,25], leaving the true magnitude of the problem underestimated precisely where it is growing fastest.

How motorcycle crashes translate into deaths depends not only on crash occurrence but on what happens after the crash. Since the classic descriptions of the temporal distribution of trauma deaths [26] and its variation across countries at different economic levels [27], a consistent body of evidence has shown that organised trauma systems, trauma-centre care, and timely emergency medical services measurably reduce mortality among the severely injured [28,29,30]. The distribution of road deaths along the care pathway—at the scene versus after reaching medical care—therefore defines where the remaining preventable mortality resides: a predominance of post-admission deaths places a substantial share of the burden, and of the opportunity, within the healthcare system itself.

Türkiye offers a striking natural setting in which these three strands converge. The registered motorcycle fleet more than doubled within seven years, coinciding with the post-pandemic expansion of the delivery economy [31], and riders came to account for a rapidly growing share of all road deaths [32]. Several forces converged to produce this expansion: the pandemic-era boom in platform delivery [14]; the affordability of small-displacement motorcycles relative to cars amid rapid currency depreciation and vehicle-price inflation; and regulatory easing, most recently the February 2024 amendment to the Road Traffic Regulation permitting holders of a class-B licence of at least two years to ride motorcycles up to 125 cm^3^ after brief certified training [33]. National assessments of Türkiye’s road-safety burden and of large-scale intervention programs exist [34,35], as do analyses of pronounced province-level disparities in road-safety performance [36] and of national cause-of-death trends [37]. The motorcycle-specific evidence base, however, remains dominated by single-centre trauma series—including recent reports of pandemic-era increases in motorcycle admissions [38] and of orthopaedic injuries among commercial couriers [39]—and by occupational studies of courier working conditions [40]; hospital-based analyses of motorcycle injury costs [41] and of in-hospital trauma mortality [42] complete the picture. No national, exposure-adjusted analysis has yet been published linking Türkiye’s fleet expansion to its motorcycle mortality and morbidity trends, the distribution of deaths along the care pathway, the occupational burden among couriers, and the anticipated demand on emergency and trauma services.

We therefore analysed official national statistics for 2018–2025 with five objectives: (i) to quantify trends in motorcycle rider deaths and injuries and their share of the national road-injury burden; (ii) to separate the contributions of exposure (fleet growth) and per-vehicle risk to the mortality increase using fleet-adjusted models; (iii) to characterise the injury burden and its care-pathway dimension—the tripling recorded-casualty caseload (operationalised as police-recorded injured persons, a proxy for emergency care demand), the change in the fatality proportion, and the distribution of deaths along the care pathway; (iv) to describe the occupational injury burden among motorcycle couriers and its registration gap; and (v) to project the trauma-care caseload to 2030 under alternative fleet scenarios. The study’s primary objective is thus quantification—of the burden, of the role of exposure, and of their distribution across the care pathway, the workforce, and the provinces; the policy and service implications developed in Section 4.4 and Section 4.6 are derived from these measurements rather than formally evaluated interventions. By tracing the burden from an emerging vulnerable occupational group to the general riding population, and from crash occurrence to trauma care, the study aims to inform injury prevention, trauma-system planning, and delivery-sector regulation in Türkiye and in other rapidly motorising settings.

## 2. Materials and Methods

### 2.1. Study Design and Setting

We conducted a national ecological study of motorcycle rider deaths and injuries in Türkiye, based entirely on routinely collected official statistics analysed at the aggregate level; no individual-level data were used, and all reported associations are ecological and descriptive rather than causal. The study period was 2018–2025, the period covered by the harmonised vulnerable road user tables of the Turkish Statistical Institute (TurkStat), with 2019–2025 as the primary window for the mortality trend models (Section 2.4); a supplementary series extending back to 2016 was used for the care-pathway analysis, and national totals were available from 2010 onward for context. The study is reported in line with the STROBE recommendations as applicable to ecological studies of routinely collected data.

### 2.2. Data Sources

Eight official or public publications were used; they derive from a smaller number of independent record-generating systems, and in particular the TurkStat casualty tables and the KGM summaries described below are two publications of the same police and gendarmerie crash records and are not independent of each other. (1) Road traffic casualty data were obtained from the TurkStat Road Traffic Accident Statistics, 2025 edition [32]. TurkStat compiles the crash reports of the police (EGM) and gendarmerie (JGK)—from the 2024 reference year onward through the Ministry of Interior’s data centre (İVME)—applies consistency checks, and publishes final figures that are not subsequently revised. Since 2015, road deaths follow the international 30-day definition: persons who die within 30 days of the crash from crash-related causes after referral to a health facility are identified in cooperation with the Ministry of Health and included. Deaths of motorcycle riders are tabulated in the vulnerable road user tables (2018–2025); injured riders, vehicle-type, province, and monthly tables from the same edition, and the counts of motorcycles involved in casualty crashes—crashes with at least one person killed or injured (Turkish ölümlü-yaralanmalı kaza)—published in TurkStat’s institutional quality reports for 2018–2025, were used for the corresponding analyses. (2) The distribution of rider deaths between the crash scene and the post-crash period (death within 30 days after referral to a health facility) was extracted from the annual traffic accident summaries of the General Directorate of Highways (KGM), which publish this breakdown by vehicle type [43]. These summaries have two compiling sources: in the 2016–2023 editions the vehicle-type tables were compiled by KGM from EGM and JGK crash data, whereas the 2025 edition reproduces TurkStat’s final figures. The EGM/JGK compilations give 7–14% fewer motorcycle-driver deaths and 9–20% fewer involved motorcycles than TurkStat’s final statistics for the same years, consistent with TurkStat’s validation and its integration of deaths within 30 days, while the 2025 KGM figures are identical to TurkStat’s (Table S3). The care-pathway series therefore contains a source break between 2023 and 2025, which is flagged wherever the series is used (Section 3.3 and Section 4.7). Full-country summaries were available for 2016–2018, 2020–2023, and 2025; the 2019 and 2024 editions are no longer published online, and these two years were treated as missing in the care-pathway series. (3) The registered motorcycle fleet was taken from the TurkStat Motor Vehicle Statistics [31]: published year-end counts were used for all study years, from 3,211,328 (2018) to 7,109,964 (2025). (4) Occupational accident data for motorcycle couriers were extracted from the statistical yearbooks of the Social Security Institution (SGK) for 2018–2025, using the economic activity class NACE H-53 (postal and courier activities) among employees insured under Article 4-1/a of Act No. 5510 [44]; complementary counts of courier deaths compiled through systematic media monitoring by a courier rights organisation were used for triangulation [45]. (5) System-level emergency medical services indicators (number of 112 emergency stations and cases per station, 2018–2023) were taken from the Health Statistics Yearbook of the Ministry of Health; annual case totals were derived as the product of the two published series [46]. (6) For external validation, transport accident deaths (ICD-10 V01–V99) registered in the TurkStat cause-of-death statistics were compared with the traffic statistics totals for the overlapping years 2022–2025 [47]; the published cause-of-death tabulation aggregates all transport accidents (V01–V99) without a road-only (V01–V89) subtotal, so this comparison is approximate and conservative (non-road transport deaths are comparatively rare); the completeness and reliability of Turkish mortality registration have been evaluated previously [48]. (7) Province-level structural covariates—hospital beds and total physicians per 100,000 population, the share of the population living in dense urban areas, gross domestic product per capita, and related socioeconomic indicators—were compiled from official national statistics (TurkStat; Ministry of Health) as period averages for 2018–2024, so that all covariates precede the 2025 outcome year. Province-level rates used the TurkStat address-based population aged 15 years and over for 2025 as the denominator. (8) As an alternative exposure denominator, the number of motorcycles covered by compulsory motor third-party liability insurance at each year-end (2018–2025) was extracted from the quarterly statistical reports of the Insurance Association of Türkiye (TSB) [49].

Vehicle definitions follow Turkish registration law (Highway Traffic Law No. 2918): ‘motorcycle’ denotes two- and three-wheeled motor vehicles, including electrically powered equivalents subject to registration, and is not limited to combustion engines. The registration statistics have no separate moped category—TurkStat defines the registered motorcycle stock as all two- and three-wheeled motor vehicles, including ATVs—so registered mopeds (motorlu bisiklet, ≤50 cm^3^) are contained in the fleet denominator [31]. In the casualty statistics, by contrast, moped drivers are tabulated as a separate and small category (in 2025, 90 deaths and 2523 injured moped drivers against 1334 and 130,578 for motorcycles) and are excluded from the rider numerators [32,43]. The fleet-adjusted rates are therefore slightly understated in level; because the moped category is small, the trends would be affected only by an abrupt change in its share of registrations, for which the casualty tables show no sign (Section 4.7). Standing e-scooters (≤25 km/h) are exempt from vehicle registration, fall outside the fleet series, and appear only in the casualty tables from 2022 onward.

### 2.3. Variables and Definitions

The primary outcomes were (i) motorcycle rider deaths and (ii) injured motorcycle riders per calendar year. Throughout, ‘rider’ denotes the person driving the motorcycle, as tabulated in the TurkStat vulnerable road user tables; pillion passengers are recorded separately in the source and are not included. The KGM care-pathway series likewise counts driver deaths by vehicle type. Reported casualties comprise deaths plus injured persons, and the provincial casualty totals include the fatalities. A supplementary table (Table S6) maps the vehicle and casualty categories of TurkStat, KGM, SGK, and TSB onto the study definitions. Secondary outcomes were the motorcycle share of all road deaths; the fleet-adjusted mortality and injury rates (per 100,000 registered motorcycles); the fatality proportion, defined as rider deaths per 100 reported rider casualties (deaths plus injured persons) and used consistently for the national series and the provincial analysis; the post-admission share of rider deaths, defined as deaths occurring within 30 days after referral to a health facility as a proportion of all rider deaths; reported occupational accidents and fatalities among couriers; and province-level casualty rates per 100,000 population aged ≥15 years. Non-motorcycle road deaths (all road deaths minus rider deaths), related to the non-motorcycle registered fleet, served as a contemporaneous comparison series indexing system-wide influences on road mortality. We deliberately avoid the term negative control: in the strict sense of Lipsitch et al. [50], a negative control outcome should be plausibly unaffected by the exposure under study, and a traffic-system aggregate does not meet this bar. The series is instead interpreted as an index of system-wide influences (enforcement, infrastructure, economic activity), and the rider and comparison trends were compared formally in a pooled model (Section 2.4); pedestrian deaths—unaffected by in-vehicle safety technology—were additionally examined as a qualitative comparator.

### 2.4. Statistical Analysis

Annual counts were modelled by Poisson regression with the natural logarithm of the registered fleet as an offset, yielding incidence rate ratios (IRRs) per calendar year for the fleet-adjusted trend [51]. The primary mortality trend was estimated for 2019–2025 because 2018—the first year of the harmonised series—showed a level difference consistent with the step change in the national total (6675 to 5473 deaths between 2018 and 2019); analyses including 2018 were run as sensitivity checks. The same specification was applied to the comparison series (non-motorcycle deaths with the non-motorcycle fleet as offset) and to injured riders. Because the injury series rose abruptly in 2021, the injured-rider trend was additionally modelled with a level shift—a flat fleet-adjusted rate to 2020, a one-off multiplicative step in 2021, and a log-linear slope thereafter—and alternative step years (2020, 2022) and a four-parameter segmented variant were compared with the single slope by the quasi-likelihood small-sample Akaike criterion (QAICc, with the overdispersion estimated from the richest model); the single-slope estimate is reported as the average annual change and interpreted alongside this model. Overdispersion was assessed by the Pearson chi-square statistic divided by the residual degrees of freedom; quasi-Poisson scaling was applied whenever this statistic exceeded unity, and the dispersion statistic, the interval method, and the Durbin–Watson statistic of the Pearson residuals are reported for every model (Table S2a,e). Negative-binomial models with moment-estimated dispersion were fitted as cross-checks. To compare the rider and non-motorcycle trends formally, the two series were also stacked in a pooled quasi-Poisson model with group, year, and group-by-year interaction terms, the interaction term estimating the ratio of the two fleet-adjusted trends. As a sensitivity analysis addressing possible denominator inflation (registered but non-circulating vehicles), all rider trend models were re-estimated with the insured fleet (compulsory motor third-party liability policies) as the offset; because the insured share of registrations changed little over time, this check is informative mainly through the stability of that share, and it is interpreted accordingly (Section 3.2). Because the final year carries the greatest leverage in a short series, and because TurkStat compiled the 2024 and 2025 statistics from a new administrative platform (İVME), all headline trend models were also re-estimated without 2025 and restricted to 2018–2023 (Table S2g). As an exploratory, data-driven change-point search—not an interrupted time-series analysis, which would require a pre-specified interruption [52,53]—segmented Poisson models with candidate level and slope changes in 2020–2023 were compared by the small-sample-corrected Akaike information criterion (AICc) against a no-break log-linear model; because the break point is selected by fit to the same data, the nominal comparison is optimistic, and given the high risk of overfitting with eight annual observations and multiple candidate breakpoints, these mortality models are reported in the Supplementary Materials only and are not interpreted substantively; for injuries, where the 2021 step is the dominant feature of the series, the level-shift model described above is reported in the main text. The contribution of exposure growth to the absolute mortality increase was quantified by decomposition: expected deaths were calculated by applying the 2021 fleet-adjusted mortality rate—2021 being the last year before the steep fleet expansion—to the observed fleet in each subsequent year, and the observed increase was compared with the increase expected from fleet growth alone; sensitivity analyses repeated the decomposition using every single-year baseline 2018–2022 and multi-year mean rates. Because the decomposition applies fitted or observed rates to observed fleets, it is an arithmetic restatement of the rate series rather than an independent estimate, and is presented as such. The fatality-proportion trend was modelled by quasi-binomial regression (dispersion-scaled). For the care pathway, the post-admission share was modelled by quasi-binomial regression on the eight available years, and—because a share can rise while both component rates fall—the scene-death and post-admission-death rates per registered motorcycle were additionally modelled separately by quasi-Poisson regression; the two unpublished years were treated as missing. Province-level inequality was summarised by the ratio of the highest to the lowest provincial rate together with the 90th/10th percentile and interquartile ratios, which are less sensitive to extreme provinces. In a cross-sectional ecological analysis of the 81 provinces, road-crash fatality proportion in 2025 (deaths per 100 reported casualties) was modelled by quasi-Poisson regression with the number of deaths as the outcome and the natural logarithm of total casualties as an offset; standardised period-average covariates (hospital beds per 100,000, physicians per 100,000, dense-urban population share, and log gross domestic product per capita) yielded rate ratios per one standard deviation, with robustness examined by adding NUTS-1 region fixed effects and by excluding the three metropolitan provinces (İstanbul, Ankara, İzmir). The share of deaths occurring after arrival at medical care was analysed analogously. A province-level association was considered robust according to an a priori criterion: preservation of direction with a 95% confidence interval excluding unity in all three specifications (main model, region fixed effects, and metropolitan exclusion). Collinearity was assessed by variance inflation factors; univariable models were fitted alongside the mutually adjusted model; and two specification checks were run—a quasi-binomial model of deaths among casualties (with standard errors scaled by the Pearson dispersion) and a complementary population-denominator model (deaths per 100,000 population aged ≥15, log population offset), the latter capturing total mortality burden rather than severity conditional on a crash. Residual spatial correlation was additionally addressed by NUTS-1 region-clustered standard errors, and province-level estimates are displayed with exact 95% confidence intervals (Figure S1) to make their uncertainty explicit. Because province-level casualty tables are not disaggregated by vehicle type, the provincial analysis refers to all road crashes and is interpreted at the population level only. Finally, the 2030 trauma caseload was projected under three fleet scenarios (compound annual growth of 4%, 8%, and 12%). The primary projections extrapolate the fitted fleet-adjusted trend models, with 95% prediction intervals obtained by Monte Carlo simulation (20,000 draws propagating parameter uncertainty and quasi-Poisson observation noise); projections holding 2025 rates constant are reported for comparison, and a sensitivity projection extrapolates the post-2021 slope of the injury level-shift model instead of the single slope (Table S5-II). Extrapolating an eight-year series five years forward assumes persistence of current trends and no saturation of fleet growth; all projections are planning scenarios, not forecasts. Analyses used Python 3.11 (Python Software Foundation, Beaverton, OR, USA) with the statsmodels library (version 0.14), and the figures were drawn with Matplotlib (version 3.10); 95% confidence intervals are reported throughout. The complete analysis code (Code S1, provided both as a Jupyter notebook and as a plain Python script) and the compiled analysis datasets (Data S1: national series; Data S2: province-level data; Data S3: crash-involvement series—TurkStat, with the EGM/JGK compilation for comparison; Data S4: insured-fleet, care-pathway, and courier-denominator series) are provided as Supplementary Materials; running the code on the supplied datasets reproduces every estimate, table, and figure reported in this article. Given the aggregate nature of the data, no imputation was performed for the two missing care-pathway years.

### 2.5. Ethics

The study used exclusively publicly available, fully anonymised, aggregate official statistics; no individual-level data or human participants were involved. Ethical review and approval were therefore not applicable under Turkish regulations on non-interventional research and the journal’s policy for studies not involving humans or animals.

## 3. Results

### 3.1. National Trends in Rider Deaths and Injuries

**Figure 1:**
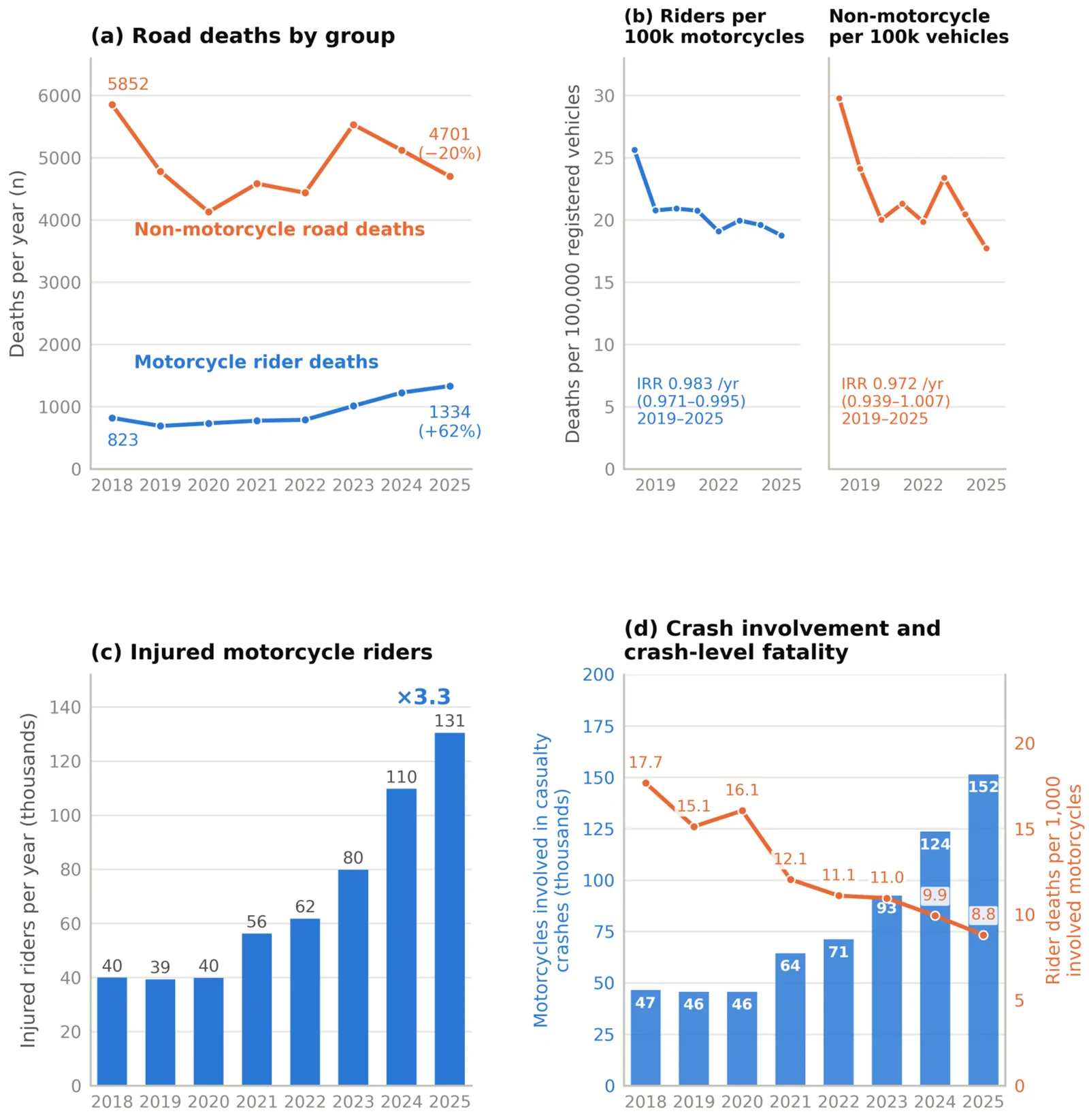
Motorcycle rider deaths and injuries in Türkiye, 2018–2025. (**a**) Road deaths by group; (**b**) fleet-adjusted mortality per 100,000 registered vehicles of each type, with the 2019–2025 trend estimates (incidence rate ratio (IRR) per year, 95% confidence interval (CI)); (**c**) injured motorcycle riders; (**d**) motorcycles involved in casualty crashes (bars; Table 2) and rider deaths per 1000 involved motorcycles (line, right axis). Sources: TurkStat [31,32].

Between 2018 and 2025, motorcycle rider deaths in Türkiye rose from 823 to 1334 per year—a 62% increase overall, and a 93% increase from the 2019 minimum of 692—while deaths among all other road users declined from 5852 to 4701 (−20%) (Table 1, Figure 1a). The motorcycle share of all road deaths consequently rose from 12.3% to 22.1%, making riders the fastest-growing group of road deaths in the country. Injured riders increased more than threefold, from 39,975 to 130,578 per year—equivalent to approximately 358 recorded injured riders per day, or one every four minutes, alongside 3.7 rider deaths per day, in 2025 (Table 1, Figure 1c). Over the same period, the registered motorcycle fleet grew 2.2-fold, from 3.21 million to 7.11 million. External validation supported the reliability of the casualty series: transport-accident deaths (ICD-10 V01–V99) in the national cause-of-death registry differed from the traffic-statistics totals by 2.7% (2022), 7.9% (2023), 0.5% (2024), and 6.4% (2025); exact agreement is not expected, because the registry publishes only the all-transport aggregate (V01–V99, including non-road modes) and tabulates by place of residence and date of death, whereas traffic statistics use the crash location and the 30-day rule. The largest gap (2023) is consistent with these tabulation differences, and both series move in the same direction throughout. The −18% step in the national total between 2018 and 2019 (6675 to 5473) affected motorcycle and non-motorcycle deaths near-proportionally (−16% and −18%) and was confined to the death series—recorded injured riders were essentially unchanged (39,975 to 39,303, −1.7%)—so 2018-anchored injury comparisons are not affected; we identified no documented methodological change in the statistical releases for 2019, and Section 2.4 describes how the analysis windows accommodate it.

Crash occurrence rose in parallel with casualties. Motorcycles involved in casualty crashes (crashes with at least one person killed or injured) increased 3.3-fold, from 46,545 in 2018 to 151,563 in 2025, closely tracking the injured-rider count (1.13–1.16 involved motorcycles per injured rider in every year), while rider deaths per 1000 involved motorcycles halved, from 17.7 to 8.8 (Table 2, Figure 1d); the trajectory of this crash-level fatality indicator mirrors the person-level fatality-proportion trend reported in Section 3.3. Both columns of Table 2 follow TurkStat definitions and are therefore internally consistent; the police-compiled (EGM/JGK) figures published in the 2016–2023 KGM editions, which are 9–20% lower for involved motorcycles and 7–14% lower for rider deaths, are shown for comparison in Table S3 (Section 2.2).

### 3.2. Exposure Versus Per-Vehicle Risk

**Figure 2:**
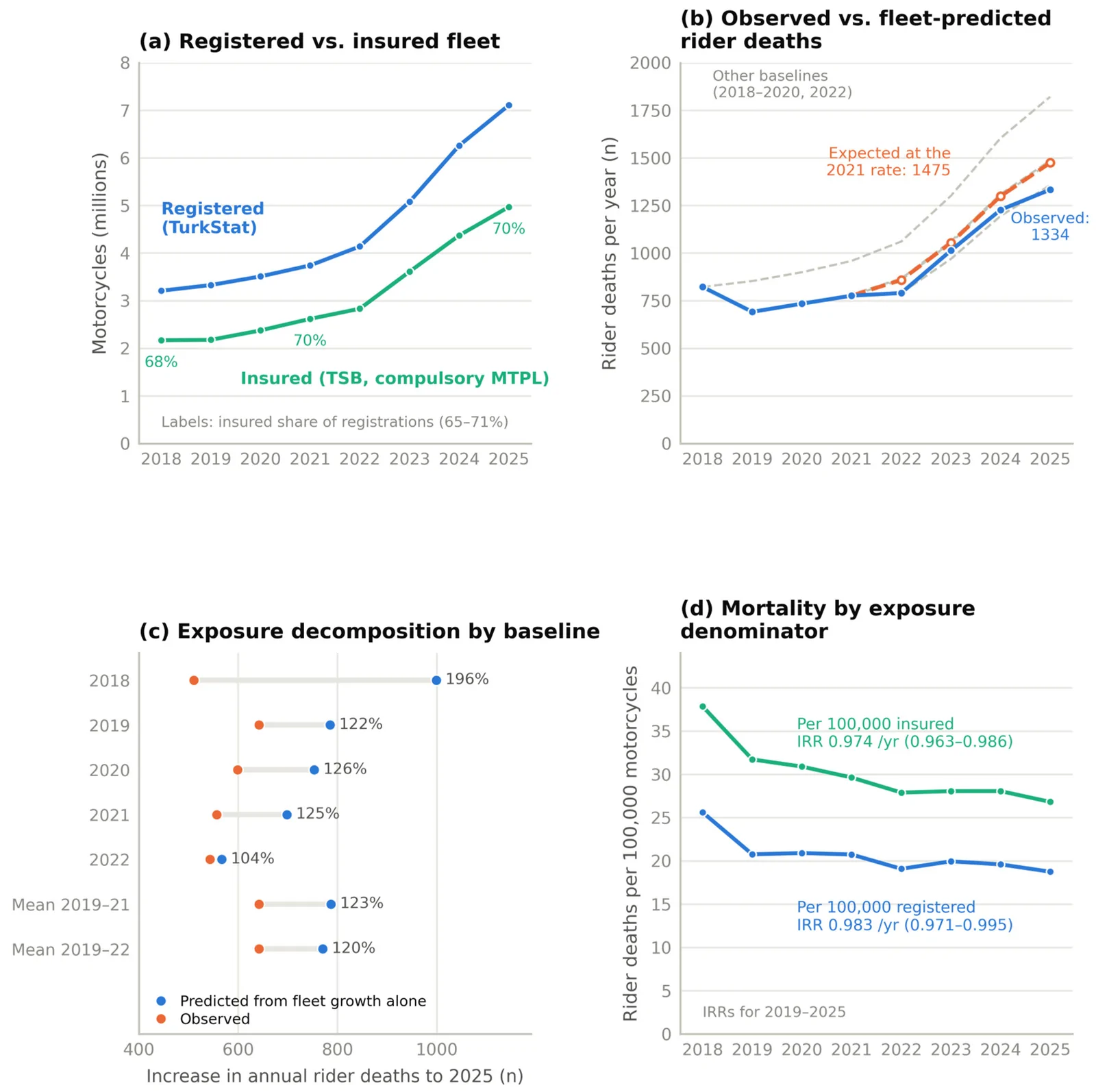
Exposure versus per-vehicle risk. (**a**) Registered motorcycle fleet (TurkStat; blue) and motorcycles under compulsory third-party liability insurance (TSB; green), with the insured share of registrations; (**b**) observed rider deaths (blue) and the deaths expected had the 2021 fleet-adjusted rate persisted (orange, dashed), with the corresponding paths for the other baselines (grey); (**c**) exposure decomposition: increase in annual rider deaths from each baseline to 2025 as observed (orange) and as predicted from fleet growth alone (blue), with the predicted/observed ratio (Table S2d); (**d**) rider deaths per 100,000 registered (blue) and per 100,000 insured (green) motorcycles with their 2019–2025 trend estimates (Table 3). Sources: TurkStat [31,32]; TSB [49].

**Figure 3:**
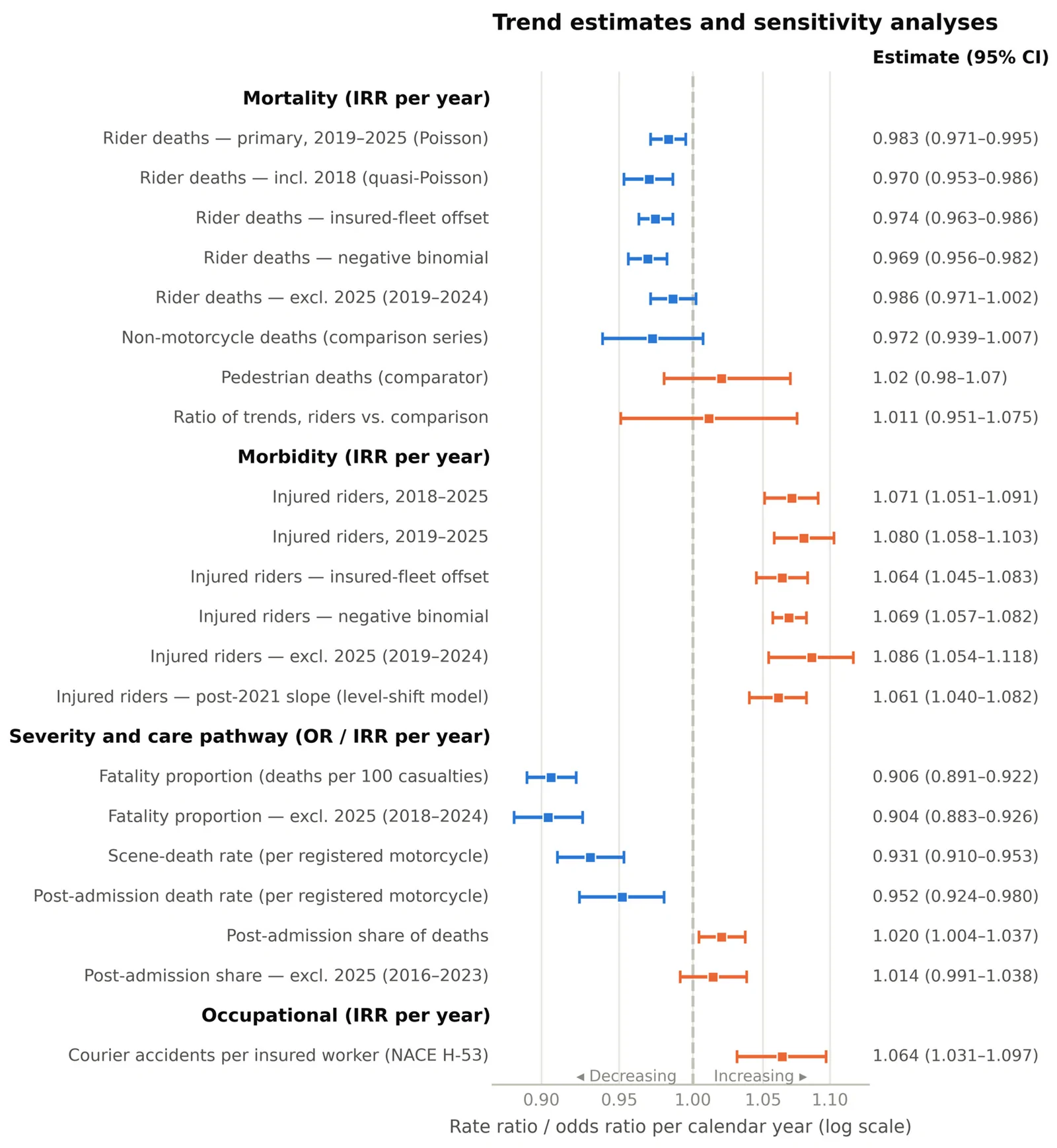
Summary of trend estimates and sensitivity analyses. Incidence rate ratios (IRR) or odds ratios (OR) per calendar year with 95% confidence intervals (log scale; dashed line: no change). Offsets and estimation windows as indicated; quasi-Poisson scaling applied where the Pearson dispersion exceeded unity, and quasi-binomial standard errors dispersion-scaled (full outputs in Table S2a,e). The ratio of trends is the pooled-model interaction term comparing the rider and non-motorcycle series. Rows labelled ‘excl. 2025’ re-estimate the model without the final year (Table S2g); the post-2021 slope is the annual change after the 2021 level shift in the injury series (Table S2b-II).

The fleet-adjusted mortality rate declined over the study period: rider deaths per 100,000 registered motorcycles fell from 25.6 in 2018 to 18.8 in 2025 (−27%), and remained in a narrow 19–21 band from 2019 through 2024, easing to 18.8 in 2025 (Figure 1b). In the primary model (2019–2025), the fleet-adjusted trend was IRR 0.983 per year (95% CI 0.971–0.995; Pearson dispersion 0.58, so Poisson scaling was retained)—a decline of about 1.7% per year; a sensitivity analysis including 2018 was consistent (IRR 0.970, 95% CI 0.953–0.986, quasi-Poisson) (Table 3). The point estimate was stable when the final year was omitted (2019–2024: IRR 0.986, 95% CI 0.971–1.002) and when the window was restricted to 2018–2023 (IRR 0.959, 95% CI 0.929–0.989), although with one or two fewer observations the 2019-anchored interval widens to include unity (Table S2g). The comparison series declined at least as fast: non-motorcycle deaths per non-motorcycle vehicle fell 40% from 2018 and 26% over the primary window (29.8 → 24.1 → 17.7 per 100,000; quasi-Poisson IRR 0.972, 95% CI 0.939–1.007), against 27% and 10% for the rider series over the same two windows—motorcycles are not outperforming the rest of the traffic system. A pooled model comparing the two series formally found no measurable difference between their fleet-adjusted trends (ratio of trends 1.011 per year, 95% CI 0.951–1.075, *p* = 0.72)—an interval that is also compatible with the comparison series improving up to about 5% per year faster than the rider series. Pedestrian deaths, examined as an additional comparator free of vehicle-technology trends, showed no significant trend over the primary window (1272 in 2019 to 1205 in 2025; quasi-Poisson IRR 1.02 per year, 95% CI 0.98–1.07). Substituting the insured fleet as the exposure denominator gave a mortality IRR of 0.974 (95% CI 0.963–0.986) for 2019–2025 and an injury IRR of 1.064 (95% CI 1.045–1.083) (Table 3; Figure 2a,d). This is not an independent confirmation: the insured share of registrations changed only slowly—67.7, 65.4, 67.7, 70.0, 68.4, 71.1, 69.8, and 69.9% in 2018 through 2025, a log-linear drift of +1.0% per year—so the insured-fleet estimate is close to the registered-fleet estimate divided by that drift (0.983/1.010 = 0.973). The informative result is the stability of the share itself: the choice of denominator can move the trend by at most about 1% per year, and in the direction of a faster decline, which makes the registered fleet the conservative denominator (Table S2a). Negative-binomial cross-checks were consistent (deaths 2018–2025: IRR 0.969, 95% CI 0.956–0.982; injuries: IRR 1.069, 95% CI 1.057–1.082); all trend and sensitivity estimates are summarised graphically in Figure 3. Exploratory change-point models for mortality are reported in the Supplementary Materials only (Table S2b); the best segmented model did not improve on a no-break linear trend (AICc 93.2 versus 93.6), and these models are not interpreted substantively (the injury series, where a level shift is the dominant feature, is treated differently in Section 3.3). Decomposition attributed the entire absolute mortality increase to exposure growth: applying the 2021 fleet-adjusted rate to the observed fleet predicted an increase of 698 annual deaths by 2025, compared with the 557 actually observed (125%) (Figure 2b). Across all baselines examined (single years 2018–2022 and multi-year means), fleet growth alone predicted 104–196% of the observed increase—that is, it consistently predicted at least the entire observed rise, over-predicting it under most baselines precisely because the fleet-adjusted rate itself declined (Figure 2c; Table S2d). As noted in Section 2.4, this decomposition restates the fitted rate series arithmetically; it quantifies, rather than independently tests, the dominance of exposure growth.

### 3.3. Injury Burden and the Care Pathway

**Figure 4:**
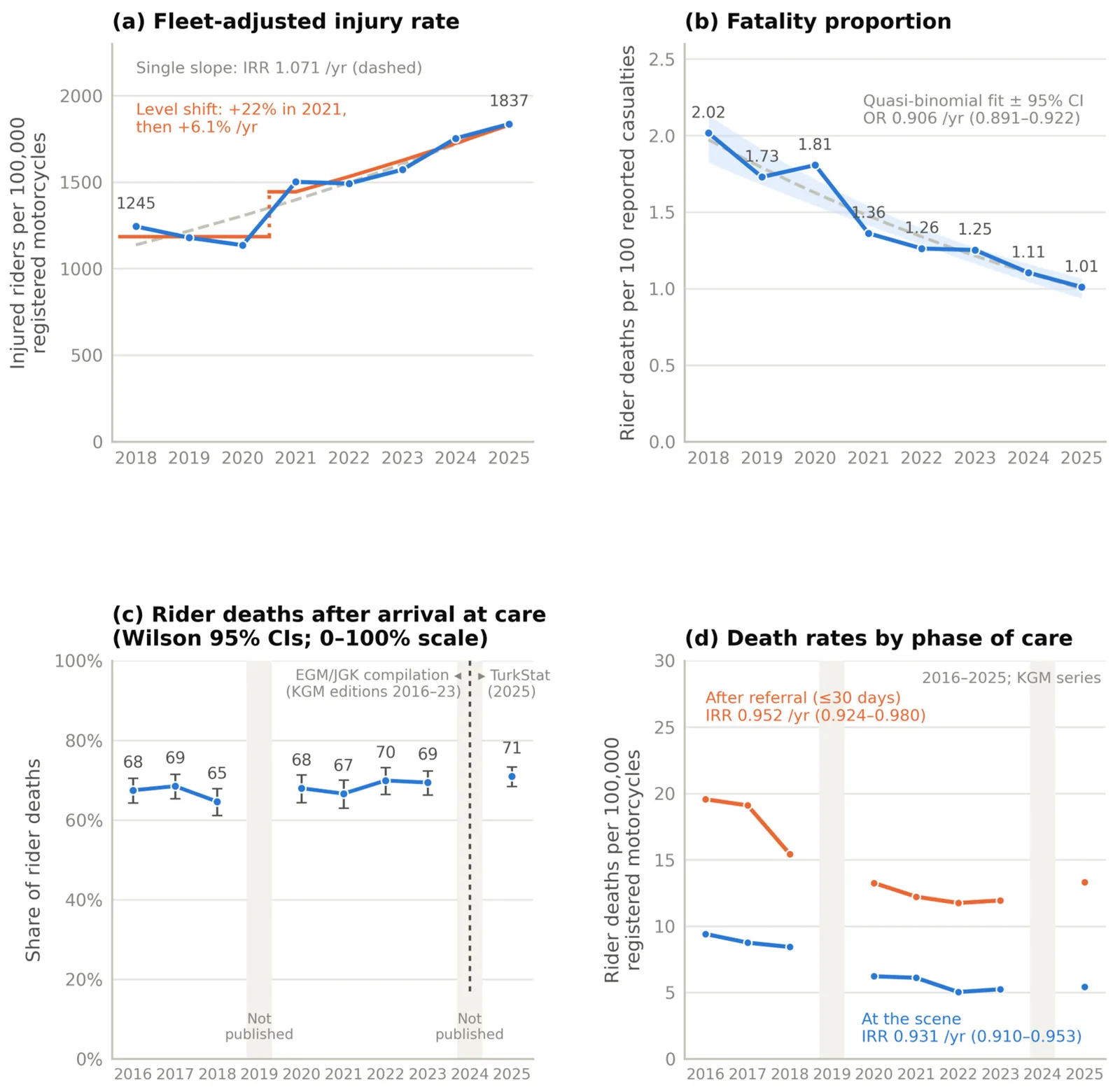
Injury burden and care pathway, 2016–2025. (**a**) Fleet-adjusted injury rate (blue) with the single-slope fit (grey, dashed) and the level-shift fit (orange; flat to 2020, step in 2021, slope thereafter; Table 3); (**b**) fatality proportion (rider deaths per 100 reported casualties; blue) with the quasi-binomial trend (grey, dashed) and its 95% confidence band (blue shading); (**c**) share of rider deaths after arrival at medical care, with Wilson 95% CIs (2019 and 2024 unpublished; the vertical line marks the change of compiling source in the KGM series, Section 2.2); (**d**) death rates at the scene (blue) and after admission (orange) per 100,000 registered motorcycles (KGM series); in (**c**,**d**) the grey vertical bands mark the two years for which no KGM edition was published (2019 and 2024). Sources: TurkStat [31,32]; KGM [43].

In contrast to mortality, the injury burden grew faster than the fleet. The fleet-adjusted injury rate rose from 1245 to 1837 injured riders per 100,000 registered motorcycles between 2018 and 2025 (+48%; quasi-Poisson IRR 1.071 per year, 95% CI 1.051–1.091; 1.080, 1.058–1.103, for 2019–2025) (Table 3). These single-slope estimates are average annual changes: the Pearson dispersion of the injury models (250 and 209) far exceeds what Poisson variation can produce for counts of this size and reflects genuine year-to-year variation around any smooth trend, and the growth was not even—the rate changed by −5.2%, −3.7%, +32.2%, −0.6%, +5.4%, +11.5%, and +4.7% in successive years. A level-shift model describes this pattern better than a single slope (QAICc 20.3 versus 32.4; Table S2b-II): the fleet-adjusted injury rate was flat through 2020, rose by 22% in 2021 (95% CI 13–32%), the year of the pandemic-era delivery expansion, and increased by 6.1% per year thereafter (95% CI 4.0–8.2%) (Table 3; Figure 4a). The injury trend was unchanged without 2025 and in the 2018–2023 window (2019-anchored IRR 1.086 and 1.085; Table S2g). The fatality proportion halved, from 2.02 to 1.01 rider deaths per 100 reported casualties (quasi-binomial OR 0.906 per year, 95% CI 0.891–0.922; 0.904, 0.883–0.926, without 2025) (Figure 4b)—a combination consistent with a change in the national crash profile—by which we mean the distribution of crashes by severity and, implicitly, by speed regime and road environment—towards lower-severity collisions; the public tables disaggregate neither collision configuration nor cause by vehicle category, and do not grade injury severity, so the profile is characterised through severity and location proxies and this interpretation is indirect (Section 4.3). Within the care pathway, death rates declined in both phases relative to the fleet—deaths at the scene fell from 9.4 to 5.4 per 100,000 registered motorcycles between 2016 and 2025 (quasi-Poisson IRR 0.931 per year, 95% CI 0.910–0.953) and post-admission deaths from 19.6 to 13.3 (IRR 0.952, 95% CI 0.924–0.980; Figure 4d)—and the post-admission phase accounted for a stable majority of deaths throughout, between 64.7% and 71.0% in every published year, with 947 of 1334 deaths (71.0%) in 2025 (Figure 4c). The apparent upward drift of this share (quasi-binomial OR 1.020 per year, 95% CI 1.004–1.037) is not robust: it disappears when 2025 is omitted (OR 1.014, 95% CI 0.991–1.038; Table S2g), and it spans the change in the compiling source of the KGM series between 2023 and 2025 (Section 2.2), so we interpret the level of the share and not its trend. Both component death rates also declined when 2025 was excluded (IRR 0.910 and 0.924 for 2016–2023; Table S2g). System-level emergency care indicators, which are not motorcycle-specific, expanded over the same period: 112 emergency stations increased from 2735 (2018) to 3420 (2023), with derived annual case totals rising from approximately 5.5 to 6.6 million over the same period (peaking at 7.4 million in 2020).

### 3.4. Occupational Burden Among Motorcycle Couriers

**Figure 5:**
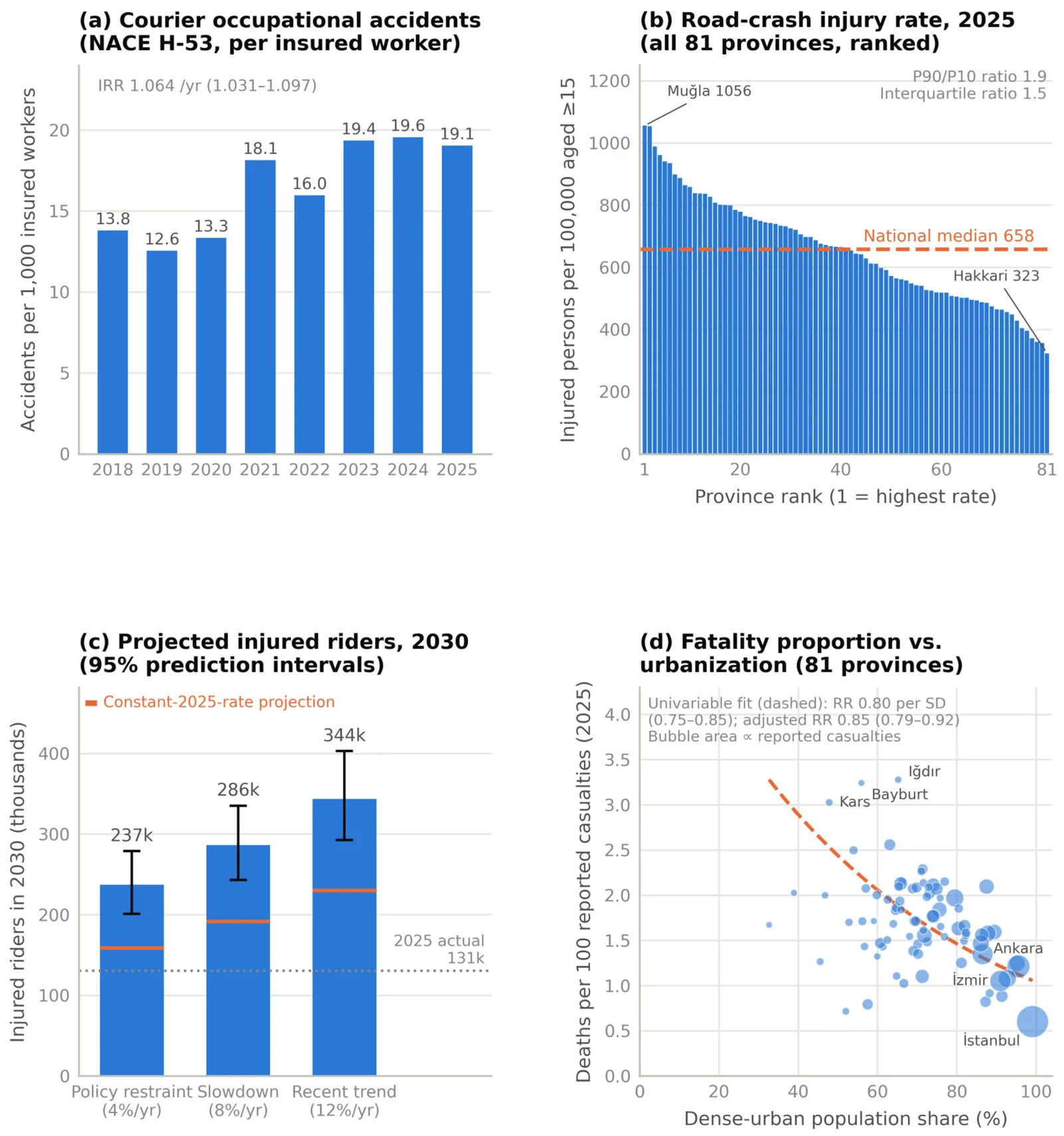
Occupational, provincial, and projection dimensions of the burden. (**a**) Courier occupational accidents per 1000 insured workers (NACE H-53; Table 4); (**b**) road-crash injuries per 100,000 population aged ≥15 years in 2025, 81 provinces ranked (dashed line: national median); (**c**) projected injured riders in 2030 under three fleet scenarios with 95% prediction intervals (orange ticks: constant-2025-rate projections; dotted line: 2025 caseload; Table S5); (**d**) provincial fatality proportion in 2025 against the dense-urban population share, with the univariable quasi-Poisson fit (dashed) and circle (bubble) area proportional to the number of reported casualties in the province (Table 5 and Table S2f). Sources: SGK [44]; TurkStat [32].

Reported occupational accidents in the postal and courier sector (NACE H-53) increased 2.7-fold between 2018 and 2025, from 1200 to 3272 per year, with the steepest single-year rise (+67%) in 2021, at the height of the pandemic-era delivery expansion (Figure 5a, Table 4). Relative to the insured workforce—which doubled from 86,914 to 171,720 over the period—the reported accident rate rose from 13.8 to 19.1 per 1000 insured workers per year (quasi-Poisson IRR 1.064, 95% CI 1.031–1.097), with a transient dip in 2022, when reported accidents fell while the insured workforce was flat—a fluctuation whose cause cannot be identified from the aggregate data (Figure 5a, Table 4). Two coverage caveats apply: H-53 accidents include non-traffic occupational injuries, so the series is not a motorcycle-crash count; and riders employed by restaurants, platform companies, or logistics firms may be registered under other activity classes (e.g., H-49 land transport, including road freight; I-56 food and beverage service activities) or work as self-employed contractors outside Article 4-1/a coverage entirely, so both the numerator and the denominator under-cover the delivery workforce. The rates should therefore be read as indicative of trend rather than as complete incidence. Officially registered occupational fatalities in the sector remained between 3 and 10 per year throughout. Independent media-monitoring counts of courier deaths were six to ten times higher in the two complete overlap years—at least 58 in 2022 and at least 68 in 2023, against 6 and 10 registered occupational fatalities, respectively (the partial 2025 media count was not used for this comparison)—indicating that the large majority of fatal courier crashes fall outside the effective coverage of the occupational-accident registry (Table 4; Section 4.5).

**Table 4:** Reported occupational accidents and fatalities in the courier sector (NACE H-53) versus media-monitored courier deaths, 2018–2025.

Year	SGK Reported Accidents	Insured Workers (H-53)	Accidents Per 1000 Insured	SGK Occupational Fatalities	Media-Monitored Courier Deaths ^1^
2018	1200	86,914	13.8	6	—
2019	1274	101,416	12.6	4	—
2020	1725	129,354	13.3	3	—
2021	2885	159,084	18.1	6	—
2022	2543	159,150	16.0	6	≥58
2023	2991	154,478	19.4	10	≥68
2024	3180	162,542	19.6	6	—
2025	3272	171,720	19.1	9	≥44 ^2^

Insured workers: employees insured under Act No. 5510 Art. 4-1/a in NACE H-53 (SGK Yearbooks 2018–2025 [44]). ^1^ Independent media-monitoring counts (Kurye Hakları Derneği [45]), published from 2022 onward; the coverage-gap comparison uses the two complete years 2022–2023. ^2^ Partial year. —: no report published.

**Table 5:** Province-level correlates of the road-crash fatality proportion, 2025 (quasi-Poisson rate ratios (RR) per +1 standard deviation (SD); 81 provinces).

Covariate (Period Average, 2018–2024)	Main Model RR (95% CI)	+NUTS-1 Region FE RR (95% CI)	Excl. İstanbul, Ankara, İzmir RR (95% CI)
Dense-urban population share	0.850 (0.789–0.916)	0.815 (0.755–0.879)	0.899 (0.845–0.956)
Physicians per 100,000	0.921 (0.854–0.993)	0.947 (0.889–1.008)	0.990 (0.905–1.083)
Hospital beds per 100,000	1.097 (1.003–1.200)	1.097 (1.025–1.175)	1.029 (0.940–1.126)
log GDP per capita	0.947 (0.898–0.999)	1.037 (0.988–1.089)	1.025 (0.969–1.084)

Outcome: road-crash deaths in 2025 (all road users; province tables are not disaggregated by vehicle type) with log casualties as offset; covariates standardised; quasi-Poisson scaling (Pearson dispersion 5.04, 2.62 and 3.52 in the three columns; *n* = 81). Univariable estimates, specification checks, variance inflation factors and region-clustered standard errors: Table S2f. FE, fixed effects.

### 3.5. Provincial Distribution of the Injury Burden

The 2025 road-crash injury burden varied 3.3-fold across provinces (90th/10th percentile ratio 1.9; interquartile ratio 1.5), from 1056 (Muğla) and 1055 (Osmaniye) to 323 (Hakkari) injured persons per 100,000 population aged ≥15 years, with the highest rates concentrated in Mediterranean, Aegean, and central Anatolian provinces (Figure 5b).

### 3.6. Projected 2030 Caseloads

Extrapolating the fitted fleet-adjusted trend models, the projected annual caseload in 2030 ranges from approximately 237,000 injured riders (95% prediction interval 201,000–279,000) and 1507 rider deaths (95% PI 1355–1676) under a policy-restraint scenario (4% annual fleet growth) to 344,000 injured riders (95% PI 293,000–403,000) and 2183 deaths (95% PI 1965–2419) if the recent 12% growth trend continues (Figure 5c, Table S5). Projections holding 2025 rates constant differ in opposite directions for the two outcomes: they are lower for injuries (159,000 to 230,000 injured riders), because they ignore the rising fleet-adjusted injury rate, and higher for deaths (1623 to 2351), because they ignore the declining mortality rate. Extrapolating the post-2021 slope of the injury level-shift model (6.1% per year) instead of the single 2019–2025 slope gives injury caseloads about 10% lower (213,000, 257,000, and 308,000 under the three scenarios; Table S5-II). All projections assume persistence of current trends without saturation and should be read as planning scenarios rather than forecasts.

### 3.7. Provincial Ecological Correlates of the Road-Trauma Fatality Proportion

Across the 81 provinces, the road-crash fatality proportion in 2025 varied more than five-fold, from 0.60 deaths per 100 reported casualties in İstanbul to 3.28 in Iğdır (90th/10th percentile ratio 2.0; interquartile ratio 1.4—extreme-value ratios overstate stable inequality); province-level estimates with exact 95% confidence intervals are displayed in Figure S1, whose extensively overlapping intervals caution against reading the ranking as a league table. Covariates showed low collinearity (all variance inflation factors ≤2.2). In univariable models, urbanisation (RR 0.80 per SD, 95% CI 0.75–0.85), physician density (0.88, 0.83–0.93), and log GDP per capita (0.91, 0.85–0.97) were each associated with a lower fatality proportion, while bed density was not (1.03, 0.93–1.13). Applying the pre-specified robustness criterion (Section 2.4), the dense-urban population share was the only covariate robustly associated with the fatality proportion: each standard-deviation increase in urbanisation corresponded to a 15% lower fatality proportion (RR 0.850, 95% CI 0.789–0.916; Figure 5d), an association that persisted with NUTS-1 region fixed effects (RR 0.815, 95% CI 0.755–0.879) and after excluding the three metropolitan provinces (RR 0.899, 95% CI 0.845–0.956) (Table 5). The association was materially unchanged in a quasi-binomial specification (OR 0.85, 95% CI 0.79–0.91), with NUTS-1 region-clustered standard errors (RR 0.85, 95% CI 0.76–0.95), and in the complementary population-denominator model (deaths per 100,000 population aged ≥15: RR 0.83, 95% CI 0.74–0.93) (Table S2f). Physician density was inversely associated with the fatality proportion in the main model (RR 0.921, 95% CI 0.854–0.993) but did not meet the robustness criterion, attenuating under region fixed effects (RR 0.947, 95% CI 0.889–1.008) and metropolitan exclusion (RR 0.990, 95% CI 0.905–1.083); this association therefore warrants cautious interpretation rather than dismissal. Hospital-bed density was positively associated with the fatality proportion in the main model (RR 1.097, 95% CI 1.003–1.200) and with region fixed effects (RR 1.097, 95% CI 1.025–1.175) but not after metropolitan exclusion (RR 1.029, 95% CI 0.940–1.126), and provincial income changed direction across specifications (RR 0.947, 95% CI 0.898–0.999; 1.037, 0.988–1.089; 1.025, 0.969–1.084); both therefore failed the criterion. The share of deaths occurring after arrival at care showed no consistent provincial correlates. The cross-sectional urbanisation gradient is consistent with the temporal pattern in Section 3.3; it is, however, equally compatible with two alternatives that the ecological data cannot separate—lower crash speeds on dense urban road networks versus intercity high-speed traffic carried by rural provinces, and more complete recording of minor-injury crashes in urban provinces, which would deflate their fatality proportions. The population-denominator model, in which the urbanisation association persists (RR 0.83), partially addresses the reporting-completeness alternative because it does not use the injury count as denominator. In the traffic statistics, deaths within 30 days are assigned to the crash province, so inter-provincial transfer of casualties to referral centres does not create a numerator–denominator mismatch, although destination-province hospital deaths still occur where care was delivered.

## 4. Discussion

### 4.1. Principal Findings

This national analysis of Türkiye’s official statistics yields five principal findings. First, motorcycle rider deaths rose by 62% between 2018 and 2025 (823 to 1334; +93% from the 2019 low of 692), raising the motorcycle share of all road deaths from 12.3% to 22.1%, while mortality in the rest of the traffic system declined. Second, this increase was exposure-driven: the fleet-adjusted mortality rate declined by about 1.7% per year (IRR 0.983, 95% CI 0.971–0.995), its trend did not differ measurably from the non-motorcycle comparison series (ratio of trends 1.011, 95% CI 0.951–1.075, an interval that also admits a somewhat faster improvement in the comparison series), the choice of exposure denominator could alter this trend by at most about 1% per year, and growth of the registered fleet alone predicted at least the entire observed increase in deaths. Third, the recorded injury burden grew disproportionately—injured riders more than tripled to over 130,000 per year; the fleet-adjusted injury rate rose by an average of 7–8% per year (IRR 1.071 for 2018–2025 and 1.080 for 2019–2025), a growth that was not even but consisted of a one-off rise of about 22% in 2021 followed by about 6% per year; and the fatality proportion halved—a pattern consistent with, though not direct evidence of, a crash profile shifting towards lower-severity collisions that generate high volumes of survivable trauma. Fourth, a stable majority of rider deaths—between roughly two-thirds and 71% in every published year, and 71% in 2025—occurred after the casualty had reached medical care, while death rates fell in both the scene and post-admission phases; the remaining mortality is thus concentrated within the trauma-care pathway. Fifth, the delivery workforce emerged as a distinct vulnerable group: reported occupational accidents in the courier sector almost tripled, while independent media-monitored courier death counts exceeded officially registered occupational fatalities six- to ten-fold—a gap that partly reflects, by construction, the occupational registry’s narrow effective coverage of this largely informal workforce.

### 4.2. An Exposure-Driven Surge in International Context

The dissociation we observed—rapidly rising absolute deaths with a flat or declining per-vehicle rate—reproduces at national scale the pattern described in rapidly motorising middle-income countries, most prominently Brazil, where the expansion of the motorcycle fleet transformed the composition of traffic mortality within two decades [9,10,11], and in Southeast Asian traffic systems [13]. Profiles of countries with rising road-death rates identify motorcycle fleet growth as their common denominator [8], and Türkiye’s recent trajectory places it firmly in this group: the fleet more than doubled in seven years while the rest of the vehicle park grew by less than half. The comparison series sharpens the interpretation. Non-motorcycle mortality per vehicle fell 26% over the primary window (40% from the 2018 anchor), against 10% (27%) for motorcycles—a difference within sampling variation (ratio of trends 1.011, 95% CI 0.951–1.075)—the motorcycle system is not deteriorating, but it shows no sign of outpacing the rest of the road network, and every increment of fleet growth converts this near-stagnant per-vehicle risk into additional absolute deaths. The policy implication follows directly: interventions that address only rider behaviour target the component of the problem that is not growing, whereas the growth itself is generated by exposure—the number of motorcycles in circulation and the intensity of their use [8,12].

### 4.3. The Changing Crash Profile and the Delivery Economy

Three converging observations link the post-2020 expansion to the delivery economy. Temporally, the steepest fleet growth, the surge in courier occupational accidents (+67% in 2021 alone), and the one-off 22% rise in the fleet-adjusted injury rate in 2021 (Section 3.3) coincided with the pandemic-era boom in platform food and parcel delivery [14]. Compositionally, the halving of the fatality proportion alongside a 50% rise in the fleet-adjusted injury rate is consistent with marginal crashes that are disproportionately urban and low-speed—the operating environment typical of delivery riding—although severity is not directly recorded, improved injury-crash recording could contribute to the same pattern (see the final paragraph of this section), and this reading remains one hypothesis among several. Spatially, the provincial cross-section showed urbanisation to be the only robust correlate of a lower fatality proportion, mirroring in space what the delivery era produced in time. This profile matches the international courier literature: emergency department series and cohorts from Australia and Korea document high crash frequency with predominantly survivable injuries among delivery riders [18,19,20], and occupational studies consistently identify time pressure, per-drop payment, incentive schedules, and fatigue as mechanisms of elevated risk [21,22,23]. Turkish single-centre series report the same signature—increased motorcycle admissions since the pandemic and heavy orthopaedic injury loads among commercial couriers [38,39]—and courier-focused occupational research in Türkiye links working conditions to safety outcomes [40]. Our national data bind these local observations into a single epidemiological picture.

The drivers of the fleet expansion itself deserve emphasis, because they define the exposure that prevention must manage. Beyond the pandemic-era surge in platform delivery [14], small-displacement motorcycles became the default affordable motorised transport as currency depreciation and vehicle-price inflation priced many households out of car ownership; and access barriers fell progressively, culminating in the February 2024 regulatory amendment allowing experienced class-B licence holders to ride motorcycles up to 125 cm^3^ after brief certified training [33]. The timing is informative but not decisive: proportional fleet growth accelerated to 22.6% in 2023, a year before the amendment, reached 23.3% in 2024—the largest absolute increment of the period, approximately 1.2 million net registrations, on a larger base—and slowed to 13.5% in 2025, the first full year with the amendment in force; the amendment thus reinforced an expansion that was already under way rather than initiating it. Fleet growth of this kind is not an exogenous force but the product of identifiable economic and regulatory choices—which is precisely why exposure management belongs on the policy agenda.

Two candidate mechanisms can produce the observed combination of a tripling injury count, a rising fleet-adjusted injury rate, and a halving fatality proportion, and they are not mutually exclusive: a genuine compositional shift towards lower-severity crashes, and improving completeness of police injury-crash recording. Injury reporting is universally less complete than fatality reporting and typically improves with electronic reporting systems and insurance-linked incentives; unlike the death series, the injury series has no external validation counterpart, and police injury counts are not severity-graded, so the two mechanisms cannot be separated with the available data. We found no documented change in police injury-recording procedures over 2018–2025, but gradual improvement cannot be excluded. The 112 emergency network expanded by roughly a quarter over the study period, and better pre-hospital reach could likewise raise recorded injuries independently of any change in crash risk. The two mechanisms cannot be separated with the available data: the compositional-shift reading remains one hypothesis among several, the injury-rate trend should be read as an upper bound on the true change in crash-generated morbidity, and corroboration against hospital or emergency-department series—once motorcycle-specific admissions data become available nationally—is the natural next step.

### 4.4. The Care Pathway: Implications for Trauma Care

In 2025, 947 of 1334 rider deaths (71%) occurred after arrival at medical care, and the share has been roughly two-thirds or more in every published year since 2016 (Section 3.3). Two readings of this finding are complementary rather than competing. Optimistically, an expanding emergency system—112 stations grew by a quarter between 2018 and 2023—delivers most critically injured riders to hospital alive, so that the post-crash phase, rather than the scene, holds the larger share of deaths. Soberly, however, the majority of deaths now occur in the phase where trauma-system performance operates: the classic literature on trauma death distributions [26,27] and the consistent mortality advantage of organised trauma systems and trauma-centre care [28,29], including the sensitivity of crash mortality to emergency response intervals [30], imply that part of a mortality burden concentrated in the post-admission phase is amenable to trauma-system performance—although this literature concerns individual-level trauma care, and an aggregate share cannot itself establish the magnitude of in-hospital preventable mortality. Importantly, both the scene and post-admission death rates declined relative to the fleet over 2016–2025; the persistently high post-admission share therefore does not signal deteriorating hospital outcomes—it locates, rather than enlarges, the remaining opportunity. The five-fold provincial gradient in the fatality proportion—an all-road-crash indicator rather than a motorcycle-specific one (Section 3.7), highest in sparsely urbanised provinces—identifies where post-crash care improvements would yield the greatest returns, while the tripling injury caseload defines the volume challenge: approximately 358 riders per day are now recorded injured nationwide—a caseload that ultimately presents to emergency and trauma services—with trend-based projections of approximately 237,000–344,000 annually by 2030 (Section 3.6). Trauma-care capacity planning, regionalised referral, and rehabilitation services therefore belong alongside crash prevention in any national response [28,29]. The geography of the emergency network matters here: Türkiye’s 112 system is planned around differential urban and rural response-time targets and its station network expanded from 2735 to 3420 between 2018 and 2023 [46], but station density and response times are not published by district or terrain type. The provincial pattern documented in Section 3.7—the highest fatality proportions in sparsely urbanised provinces, where distances are long, intercity high-speed roads dominate, and winter conditions in mountainous districts can slow both response and transport—is consistent with pre-hospital reach in rural and mountainous areas being the binding constraint, and area-stratified EMS response-time reporting would allow this to be tested directly.

### 4.5. Couriers: An Emerging Occupational Group and a Registration Gap

The gap between media-monitored courier deaths and officially registered occupational fatalities is, in part, a coverage mismatch by construction: the media counts enumerate all couriers—self-employed, informal, and platform-contracted riders included—whereas the SGK series covers only employees insured under Article 4-1/a within one activity class. The two numerators therefore describe different populations, and the six- to ten-fold ratio should be read as a measure of how narrow the occupational registry’s effective coverage of this workforce is, not as an under-reporting rate within its scope. The media counts themselves are conservative minimums [45]. Beyond this definitional mismatch, the remaining gap is plausibly explained by the structure of platform work: riders operating as self-employed contractors, informally employed, or registered outside the courier activity class fall outside the occupational-accident registry’s effective coverage, a pattern documented internationally for precarious and gig work [15,16,24] and for occupational injury under-reporting generally [25,54]. Capture–recapture studies of fatal occupational injuries demonstrate that even official death registries miss a substantial share of work-related deaths when employment status is ambiguous [55]; a formal capture–recapture estimate for Turkish couriers would require case-level identification of the overlap between the media-monitored and SGK sources, which the published aggregates do not permit, and is an important target for future record-linkage work. The consequence is not merely statistical: unregistered occupational deaths generate no employer accountability, no occupational-safety inspection trigger, and no survivor benefits, and they exclude the courier workforce from the risk-based prevention apparatus of occupational health [15,25]. Formal recognition of motorcycle delivery as a high-risk occupation, platform-level incident reporting obligations, and linkage of traffic and social-security records would close much of this gap.

### 4.6. Policy Implications

The findings assemble into a three-tier agenda aligned with the Safe System approach [56] and the WHO Decade of Action targets [6,7]. The agenda is inherently intersectoral: crash risk is produced by road-network design, traffic and licensing policy, and labour-market incentives as much as by individual behaviour, so motorcycle-injury prevention is a cross-sectoral governance task—transport, labour, urban planning, and health acting together, in the spirit of Health-in-All-Policies—rather than a task for the health sector alone [6,7,56]. First, exposure management: since fleet growth drives mortality, licensing rigour for the small-displacement segment, graduated access for young and novice riders [57,58], and delivery-platform regulation—caps on algorithmic speed incentives, per-drop payment reform, and enforceable working-time limits [21,22,23]—act on the quantity and intensity of riding itself. Second, in-crash protection: helmet use remains the single most effective individual countermeasure [59,60], and enforcement experience in motorcycle-dependent middle-income systems shows rapid head-injury reductions when universal laws are enforced [61]; speed management in urban networks compounds this protection [58]. Adoption of MAIS-based injury-severity coding in the police statistics, as in current European practice, and routine linkage of crash, hospital, and social-security records would convert the surveillance gaps documented here into measurable indicators. Third, post-crash care: with roughly two-thirds to 71% of deaths occurring in the care pathway and a five-fold provincial gradient in the fatality proportion, investment in trauma-system organisation, emergency response, and rehabilitation—targeted at the sparsely urbanised, high-fatality provinces, for road trauma generally, as the provincial indicator is not motorcycle-specific—addresses the phase of injury where the remaining mortality now concentrates [28,29,30]. The projections quantify the stakes: the difference between the policy-restraint and recent-trend fleet scenarios amounts to roughly 107,000 injured riders and nearly 700 rider deaths per year by 2030 under trend-based projections.

### 4.7. Strengths and Limitations

The study’s strengths include complete national coverage over eight years under a stable 30-day death definition; cross-validation of the casualty series against the independent cause-of-death registry (differences 0.5–7.9%); explicit separation of exposure from per-vehicle risk with a contemporaneous comparison series and a formal test of trend difference; quasi-Poisson scaling of standard errors; triangulation across independent record-generating systems—police and gendarmerie crash records, vehicle-registration and compulsory-insurance registers, social-security registers, media monitoring, health-system statistics, and civil death registration—on which the eight publications used draw (TurkStat’s and KGM’s crash tables derive from the same police records; Section 2.2); and a provincial cross-section consistent with the temporal fatality-proportion pattern. Several limitations warrant emphasis. The design is ecological throughout: no individual-level information on age, helmet use, alcohol, licensure, or delivery employment was available, and the courier attribution rests on temporal and compositional coincidence rather than person-level linkage. The registered fleet is an imperfect exposure proxy—vehicle-kilometres travelled are not published for motorcycles, and the fleet’s composition shifted towards small-displacement machines whose usage intensity differs; the insured fleet offers only a partial check: because the insured share of registrations stayed within 65–71% and drifted by about 1% per year, the choice between the two denominators can move the trend estimates by at most about 1% per year (Section 3.2), which argues against a growing inflation of the registered fleet by dormant vehicles but says nothing about usage intensity. The −18% step in total road deaths between 2018 and 2019 lacks a documented methodological explanation; it coincided with the 2018–2019 economic contraction and intensified enforcement, affected motorcycle and non-motorcycle deaths near-proportionally, and was accommodated by starting the primary mortality window in 2019. All national models rest on seven or eight annual observations; Durbin–Watson statistics are reported for completeness (Table S2a), but such diagnostics have minimal power at this series length, and all trend estimates should be read with this in mind. For the injury series in particular, the single log-linear slope averages across a level shift in 2021, so we report the level-shift model alongside it (Section 3.3); the residual dispersion of even that model (83) shows that year-to-year variation, not Poisson noise, dominates counts of this size, and the quasi-Poisson intervals are descriptive bands around an average growth rate rather than exact inferential statements. The cause-of-death cross-validation relies on the published all-transport aggregate (V01–V99), as no road-only (V01–V89) subtotal is released. The injury series, which carries the morbidity argument, has no external validation counterpart, and its growth conflates true incidence with reporting completeness (Section 4.3). The registries contain no information on injury type, body region, severity, or clinical cause of death, so neither a typical injury profile nor the leading causes of death could be characterised; police-recorded casualty outcomes are binary (killed under the 30-day rule, or injured), with no MAIS-type severity grading published in the aggregate tables and no public linkage to hospital ICD-10 records, so the injury surge cannot be decomposed into severity strata; collision configurations and fault attributions are likewise published only for all vehicles combined, not by vehicle category; the care-pathway series lacks two years (2019, 2024) and, as documented in Section 2.2, was compiled from police and gendarmerie data in the 2016–2023 KGM editions but reproduces TurkStat’s final figures in the 2025 edition—a source break that we address by interpreting the post-admission share as a level rather than a trend. The 2024 and 2025 TurkStat statistics were compiled from a new administrative data platform (İVME); the headline trends were unchanged when 2025 was omitted or the window restricted to 2018–2023 (Table S2g). The registered ‘motorcycle’ fleet contains mopeds and ATVs, whose drivers are not in the rider numerators; this understates the level of the fleet-adjusted rates slightly and would affect the trends only if the small moped share of registrations had changed abruptly, for which the casualty tables, which record moped drivers separately, show no sign. SGK occupational data cover only formally insured employees, and media-monitored courier counts are minimums. The provincial analysis concerns all road crashes, not motorcycles specifically, and cross-sectional ecological associations—including the positive bed-density association, most plausibly reflecting supply following burden or referral of severe casualties towards better-equipped provinces—rest on covariates averaged over 2018–2024 and assumed stable into 2025, were protected against residual spatial clustering only through region fixed effects and region-clustered standard errors, and must not be read causally. The heterogeneous composition of the non-motorcycle comparison series limits its interpretation as a strict counterfactual (Section 2.3), although the pedestrian comparator points in the same direction. Finally, the 2030 projections extrapolate short series under assumed fleet-growth scenarios without saturation; the trend-based estimates carry wide prediction intervals, and the constant-rate variants bound the trend-based estimates from below for injuries (a rising injury rate) but from above for deaths (a declining mortality rate); extrapolating the post-2021 injury slope instead of the single slope lowers the injury projections by about 10% (Table S5-II).

## 5. Conclusions

Türkiye’s motorcycle mortality surge is exposure-driven: deaths rose by 62% between 2018 and 2025 (and by 93% from the 2019 low) not because riding became more dangerous per vehicle, but because the delivery-economy era more than doubled the number of motorcycles on the road while their per-vehicle risk stood still against an otherwise improving traffic system. The consequences for emergency and trauma care are larger than the mortality figures suggest—a tripled injury caseload approaching 360 emergency presentations per day, a fatality proportion that halved, signalling high-volume and predominantly survivable trauma, and roughly two-thirds to 71% of deaths occurring after arrival at medical care even as death rates fell in both phases of the care pathway. Prevention policy should therefore act on three fronts simultaneously: managing exposure through licensing and delivery-platform regulation, closing the occupational registration gap that leaves the courier workforce outside formal protection, and strengthening trauma and post-crash care where the remaining mortality—and its steep provincial inequality—now resides. For health services, the operational meaning is concrete: if current trends persist, emergency and trauma networks should plan for a recorded motorcycle-casualty caseload on the order of 240,000–340,000 per year by 2030—roughly 650–940 presentations per day, against about 360 today, with an upper prediction bound just over 400,000 (403,000)—and provision orthopaedic, neurosurgical, rehabilitation, and blood-product capacity accordingly; the provincial gradient argues for directing pre-hospital and post-crash care investment towards the sparsely urbanised provinces, for road trauma generally; and closing the courier registration gap would extend occupational protection to a rapidly expanding workforce. These are the actionable consequences of a burden that is growing by exposure, not by per-vehicle risk.

## Figures and Tables

**Table 1:** Motorcycle rider deaths, injuries, and fleet-adjusted rates, Türkiye, 2018–2025.

Year	Rider Deaths	All Road Deaths	% of All Road Deaths	Injured Riders	Fatality Proportion ^1^	Fleet (×1000) ^2^	Deaths/100,000 Fleet	Injuries/100,000 Fleet
2018	823	6675	12.3	39,975	2.02	3211	25.6	1245
2019	692	5473	12.6	39,303	1.73	3331	20.8	1180
2020	735	4866	15.1	39,918	1.81	3513	20.9	1136
2021	777	5362	14.5	56,257	1.36	3744	20.8	1502
2022	791	5229	15.1	61,842	1.26	4142	19.1	1493
2023	1014	6548	15.5	79,902	1.25	5079	20.0	1573
2024	1228	6351	19.3	109,832	1.11	6262	19.6	1754
2025	1334	6035	22.1	130,578	1.01	7110	18.8	1837

^1^ Fatality proportion: rider deaths per 100 reported rider casualties (deaths + injured persons). ^2^ Official year-end registered motorcycle counts. Sources: TurkStat Road Traffic Accident Statistics, 2025 edition; TurkStat Motor Vehicle Statistics.

**Table 2:** Motorcycles involved in casualty crashes and rider deaths per 1000 involved motorcycles, Türkiye, 2018–2025.

Year	Motorcycles Involved in Casualty Crashes ^1^	Rider Deaths	Deaths Per 1000 Involved Motorcycles
2018	46,545	823	17.7
2019	45,711	692	15.1
2020	45,753	735	16.1
2021	64,479	777	12.1
2022	71,270	791	11.1
2023	92,565	1014	11.0
2024	123,675	1228	9.9
2025	151,563	1334	8.8

^1^ Casualty crash: at least one person killed or injured (ölümlü-yaralanmalı kaza); one crash may involve several vehicles. Source: TurkStat Road Traffic Accident Statistics (institutional quality reports and vulnerable road user tables) [32]; both columns follow TurkStat definitions. The EGM/JGK-compiled KGM figures are compared in Table S3.

**Table 3:** Fleet-adjusted trend estimates (Poisson regression with log fleet offset).

Series	Years	IRR per Year (95% CI)	Model
Rider deaths (primary)	2019–2025	0.983 (0.971–0.995)	Poisson
Rider deaths, incl. 2018	2018–2025	0.970 (0.953–0.986)	Quasi-Poisson
Non-motorcycle deaths (comparison)	2019–2025	0.972 (0.939–1.007)	Quasi-Poisson
Injured riders	2018–2025	1.071 (1.051–1.091)	Quasi-Poisson
Injured riders	2019–2025	1.080 (1.058–1.103)	Quasi-Poisson
Injured riders, level shift in 2021: one-off step ^1^	2021 vs. 2018–2020	1.219 (1.130–1.315)	Quasi-Poisson
Injured riders, post-2021 slope ^1^	2021–2025	1.061 (1.040–1.082)	Quasi-Poisson
Rider deaths, insured-fleet offset	2019–2025	0.974 (0.963–0.986)	Poisson
Injured riders, insured-fleet offset	2018–2025	1.064 (1.045–1.083)	Quasi-Poisson
Ratio of trends, riders vs. comparison (pooled interaction)	2019–2025	1.011 (0.951–1.075)	Quasi-Poisson

Offsets: log registered motorcycles (rider series) and log non-motorcycle registered vehicles (comparison series); quasi-Poisson scaling where the Pearson dispersion exceeded unity (full outputs, Table S2a; estimates without 2025, Table S2g). Insured fleet: motorcycles under compulsory third-party liability coverage (TSB) [49]. ^1^ Level-shift model: flat rate to 2020, a one-off step in 2021 (a ratio, not per year) and a log-linear slope thereafter (QAICc 20.3 vs. 32.4 for the single slope; Table S2b-II); the single-slope injury estimates average across this step.

## Data Availability

All data used in this study are publicly available from the cited official sources (TurkStat, KGM, SGK, TSB, Ministry of Health). The complete compiled analysis datasets are provided as Supplementary Materials (Data S1–S4, CSV format), together with the full analysis code (Code S1) that reproduces all reported results.

## Supplementary Materials

The following supporting information can be downloaded at: https://www.mdpi.com/article/10.3390/healthcare14183072/s1, Table S1: province-level road-crash outcomes and period-average covariates, 81 provinces; Table S2: full model outputs, including dispersion and autocorrelation diagnostics, AICc/QAICc comparisons and the injury level-shift models, provincial specification checks, insured-fleet sensitivity models, and the leave-2025-out and 2018–2023 sensitivity estimates; Table S3: care-pathway series 2016–2025 with reconciliation of the KGM and TurkStat death counts; Table S4: data source inventory; Table S5: 2030 projections with 95% prediction intervals and the post-2021-slope sensitivity (Table S5-II); Table S6: correspondence of definitions across TurkStat, KGM, SGK, and TSB sources; Figure S1: provincial fatality proportions with 95% confidence intervals); Code S1: full analysis code (CodeS1_analysis.ipynb/CodeS1_analysis.py) and figure code (CodeS1_figures.py); Data S1: compiled national series (DataS1_national_series.csv); Data S2: compiled province-level dataset (DataS2_provinces.csv); Data S3: crash-involvement series, TurkStat with the EGM/JGK compilation for comparison (DataS3_crash_involvement.csv); Data S4: insured-fleet, care-pathway, and courier-denominator series (DataS4_insured_series.csv); STROBE checklist (STROBE_checklist.docx). Running Code S1 on Data S1–S4 reproduces all reported results.
