# Supplementary material for: The Rising Burden of Motorcycle Crashes and Rider Injuries in Türkiye (2018–2025): An Ecological Analysis of Routinely Collected National Statistics with Implications for Injury Prevention and Trauma Care
Source: Healthcare (Basel). 2026 Sep 18;14(18):3072. doi: 10.3390/healthcare14183072 (PMC13606858; doi:10.3390/healthcare14183072)
Supplement: Supplementary file 1 [file healthcare-14-03072-s001.zip › healthcare-4521589-supplementary.pdf]

Supplementary Materials

The Rising Burden of Motorcycle Crashes and Rider Injuries in Türkiye (2018–2025): An Ecological Analysis of Routinely Collected National Statistics with Implications for Injury Prevention and Trauma Care — Doğan, Parlak, Etli

Table S1. Province-level road-crash outcomes (2025) and period-average covariates (2018–2024), 81 provinces

Province	Population ≥15	Deaths	At scene	After care	Injured	Injured /100k	CFR [1]	Beds /100k	Physicians /100k	Urban %
Adana	1,781,370	198	71	127	9,246	519	2.10	324	220	87.5
Adıyaman	456,725	71	23	48	3,353	734	2.07	207	158	68.8
Afyonkarahisar	606,530	125	24	101	4,759	785	2.56	311	180	63.1
Aksaray	344,298	54	26	28	2,522	733	2.10	232	144	65.3
Amasya	287,167	40	20	20	2,702	941	1.46	255	150	70.1
Ankara	4,856,106	290	159	131	23,609	486	1.21	376	373	95.5
Antalya	2,258,185	258	98	160	18,884	836	1.35	297	237	86.5
Ardahan	74,158	5	3	2	294	396	1.67	222	162	32.6
Artvin	142,927	9	7	2	516	361	1.71	210	170	59.1
Aydın	970,523	135	59	76	8,135	838	1.63	300	201	80.4
Ağrı	342,331	25	7	18	1,721	503	1.43	173	112	61.3
Balıkesir	1,088,969	145	73	72	8,059	740	1.77	270	169	74.0
Bartın	177,146	13	6	7	1,014	572	1.27	229	141	45.5
Batman	464,515	38	4	34	2,361	508	1.58	269	133	82.3
Bayburt	68,173	12	2	10	358	525	3.24	280	171	55.9
Bilecik	189,007	24	14	10	1,583	838	1.49	233	146	81.8
Bingöl	214,986	17	7	10	1,519	707	1.11	253	137	64.8
Bitlis	256,129	27	5	22	1,440	562	1.84	291	137	66.0
Bolu	275,933	39	13	26	1,773	643	2.15	455	275	66.2
Burdur	235,051	45	19	26	2,260	961	1.95	281	158	62.6
Bursa	2,629,091	149	58	91	13,636	519	1.08	262	191	92.7
Denizli	872,780	133	63	70	6,289	721	2.07	309	212	74.9
Diyarbakır	1,301,312	109	36	73	6,053	465	1.77	272	175	74.0
Düzce	337,746	42	12	30	2,224	658	1.85	216	200	80.5
Edirne	363,574	40	18	22	1,726	475	2.27	469	325	71.0
Elazığ	486,510	53	15	38	2,411	496	2.15	525	223	76.9
Erzincan	197,694	25	9	16	1,636	828	1.51	268	203	62.5
Erzurum	570,958	57	26	31	2,888	506	1.94	483	243	65.6
Eskişehir	778,475	36	18	18	4,344	558	0.82	402	251	87.2
Gaziantep	1,585,663	157	60	97	9,698	612	1.59	335	165	89.4
Giresun	388,326	34	24	10	1,952	503	1.71	386	182	56.2
Gümüşhane	116,886	15	5	10	735	629	2.00	243	149	46.7
Hakkari	207,422	9	7	2	671	323	1.32	159	132	59.9
Hatay	1,193,257	179	54	125	11,159	935	1.58	273	161	87.8
Isparta	371,539	56	22	34	3,213	865	1.71	472	299	69.6
Iğdır	152,422	24	9	15	708	464	3.28	157	122	65.2
Kahramanmaraş	874,026	151	50	101	6,983	799	2.12	260	161	74.0
Karabük	213,437	24	14	10	1,427	669	1.65	296	201	75.9
Karaman	211,322	27	6	21	1,646	779	1.61	238	153	71.6
Kars	207,012	29	9	20	929	449	3.03	280	171	47.8
Kastamonu	325,286	51	28	23	1,991	612	2.50	304	158	53.9
Kayseri	1,156,891	124	45	79	7,821	676	1.56	333	221	86.3
Kilis	116,314	21	1	20	1,046	899	1.97	273	203	75.8
Kocaeli	1,708,954	129	37	92	10,219	598	1.25	243	183	95.2

Konya	1,842,446	275	108	167	13,679	742	1.97	370	222	79.5
Kütahya	481,887	60	22	38	3,210	666	1.83	345	175	64.5
Kırklareli	324,277	38	13	25	1,779	549	2.09	267	162	72.9
Kırıkkale	236,696	30	13	17	1,894	800	1.56	445	231	82.3
Kırşehir	201,396	22	8	14	1,406	698	1.54	223	175	76.9
Malatya	607,832	82	34	48	3,978	654	2.02	409	231	73.1
Manisa	1,203,270	172	87	85	9,173	762	1.84	327	187	75.6
Mardin	624,883	43	25	18	2,853	457	1.48	179	131	72.6
Mersin	1,553,128	174	102	72	11,697	753	1.47	266	174	86.0
Muğla	923,391	154	57	97	9,752	1056	1.55	226	198	71.8
Muş	271,785	7	4	3	970	357	0.72	188	114	52.0
Nevşehir	261,119	49	19	30	2,583	989	1.86	257	140	64.7
Niğde	297,754	51	35	16	2,406	808	2.08	250	134	57.0
Ordu	640,627	33	15	18	4,127	644	0.79	295	165	57.5
Osmaniye	433,729	58	25	33	4,574	1055	1.25	261	129	81.1
Rize	289,799	28	13	15	1,635	564	1.68	323	229	64.0
Sakarya	907,038	102	39	63	6,038	666	1.66	212	177	81.9
Samsun	1,147,830	86	36	50	7,712	672	1.10	377	237	71.2
Siirt	232,244	18	5	13	1,147	494	1.55	241	130	68.1
Sinop	192,730	21	9	12	1,444	749	1.43	301	149	56.7
Sivas	517,705	80	41	39	3,757	726	2.08	437	216	69.8
Tekirdağ	972,311	47	31	16	5,275	543	0.88	274	157	91.4
Tokat	509,718	61	26	35	4,086	802	1.47	373	173	60.6
Trabzon	679,328	49	19	30	3,579	527	1.35	408	260	70.2
Tunceli	73,555	9	2	7	435	591	2.03	175	211	38.9
Uşak	311,925	47	21	26	2,323	745	1.98	321	156	72.4
Van	788,696	59	26	33	3,384	429	1.71	267	151	69.3
Yalova	254,615	18	7	11	1,947	765	0.92	247	163	88.3
Yozgat	341,882	48	32	16	2,348	687	2.00	300	167	59.8
Zonguldak	500,007	28	10	18	2,704	541	1.02	386	198	66.6
Çanakkale	489,397	80	41	39	3,414	698	2.29	315	227	71.4
Çankırı	168,351	25	10	15	1,445	858	1.70	236	142	52.8
Çorum	433,483	54	19	35	3,846	887	1.38	321	171	69.0
İstanbul	12,808,144	289	134	155	47,717	373	0.60	289	266	99.1
İzmir	3,764,185	208	122	86	19,563	520	1.05	291	286	91.0
Şanlıurfa	1,432,216	152	52	100	6,987	488	2.13	194	120	65.8
Şırnak	384,940	34	13	21	1,557	404	2.14	130	112	71.5

[1] Fatality proportion: deaths per 100 reported casualties (deaths + injured). Outcomes: TurkStat Road Traffic Accident Statistics, 2025 edition (province tables; all road users). Covariates: period averages 2018–2024 compiled from official statistics (TurkStat; Ministry of Health).

Table S2. Full model outputs

Table S2a. National trend models (offset: log registered fleet; comparison series: log non-motorcycle fleet)

Model (series, years)	Scale (dispersion)	Term	β (SE)	IRR (95% CI)	p	DW
Rider deaths — primary, 2019–2025	Poisson (0.58)	Intercept	−8.4670 (0.0247)	—	<0.001	
		Year	−0.0171 (0.0061)	0.983 (0.971–0.995)	0.006	
Rider deaths — incl. 2018, 2018–2025	Quasi-Poisson (3.04)	Intercept	−8.3757 (0.0402)	—	<0.001	
		Year	−0.0309 (0.0087)	0.970 (0.953–0.986)	<0.001	
Non-motorcycle deaths — comparison, 2019–2025	Quasi-Poisson (42.78)	Intercept	−8.3858 (0.0657)	—	<0.001	
		Year	−0.0283 (0.0179)	0.972 (0.939–1.007)	0.114	
Injured riders, 2018–2025	Quasi-Poisson (250.16)	Intercept	−4.4756 (0.0479)	—	<0.001	

		Year	+0.0687 (0.0096)	1.071 (1.051–1.091)	<0.001	
Injured riders, 2019–2025	Quasi-Poisson (209.27)	Intercept	−4.4462 (0.0449)	—	<0.001	
		Year	+0.0773 (0.0104)	1.080 (1.058–1.103)	<0.001	
Rider deaths — insured-fleet offset, 2019–2025	Poisson (0.38)	Year	−0.0259 (0.0062)	0.974 (0.963–0.986)	<0.001	1.66
Rider deaths — insured-fleet offset, 2018–2025	Quasi-Poisson (2.40)	Year	−0.0386 (0.0078)	0.962 (0.948–0.977)	<0.001	1.29
Injured riders — insured-fleet offset, 2018–2025	Quasi-Poisson (221.2)	Year	+0.0616 (0.0090)	1.064 (1.045–1.083)	<0.001	2.39
Pooled riders vs. comparison, 2019–2025	Quasi-Poisson (21.7)	Group × Year	+0.0112 (0.0313)	1.011 (0.951–1.075)	0.72	—
Rider deaths — negative binomial (α=0.0010), 2018–2025	NB2	Year	−0.0317 (0.0069)	0.969 (0.956–0.982)	<0.001	—
Injured riders — negative binomial (α=0.0015), 2018–2025	NB2	Year	+0.0669 (0.0060)	1.069 (1.057–1.082)	<0.001	—

β: log-scale coefficient; SE: standard error; IRR: incidence rate ratio per calendar year; DW: Durbin–Watson statistic of the Pearson residuals (reported for the year term of each series; values above 2 — up to 3.0 for the injury models — point to negative rather than positive first-order serial correlation; with n = 7–8 annual observations the statistic has minimal power and is reported for completeness only). Insured share of the registered fleet used in the insured-fleet models: 67.7% (2018), 65.4 (2019), 67.7 (2020), 70.0 (2021), 68.4 (2022), 71.1 (2023), 69.8 (2024), 69.9 (2025); because this share drifted by only about +1.0% per year (log-linear, 2019–2025), the insured-fleet mortality IRR (0.974) is close to the registered-fleet IRR divided by that drift (0.983/1.010 = 0.973) and is not an independent estimate — the informative result is the stability of the share, which bounds the effect of the denominator choice on the trend at about 1% per year. Quasi-Poisson scaling applied whenever the Pearson dispersion exceeded unity. Insured fleet: motorcycles under compulsory motor third-party liability coverage (TSB). Negative-binomial dispersion parameters estimated by the method of moments. All intervals are Wald intervals on the log scale (estimate ± 1.96 SE), with SEs scaled where quasi-scaling applied.

Table S2b. Exploratory segmented Poisson models versus the no-break trend (level + slope change; offset: log registered fleet; 2018–2025, *n* = 8)

Model	AIC	AICc	Level-change IRR	Slope-change IRR (per year)	Dispersion
No-break linear trend	91.2	93.6	—	—	3.04
T = 2020	79.9	93.2 [1]	1.237	1.210	0.68
T = 2021	85.6	98.9	1.119	1.089	2.09
T = 2022	88.8	102.1	1.050	1.055	2.90
T = 2023	87.0	100.3	1.124	1.029	2.45

[1] Lowest AICc among segmented models; it does not improve on the no-break trend (ΔAICc = 0.4). This table concerns the mortality series; the injury series, where a level shift is the dominant feature, is analysed in Table S2b-II. AICc: small-sample-corrected Akaike information criterion, AIC + 2k(k+1)/(n−k−1). With eight observations and four parameters, segmented models are reported for transparency only and are not interpreted substantively; because the break point is selected by fit to the same data, even the AICc comparison is optimistic for the segmented models.

Table S2b-II. Injured riders, 2018–2025: single log-linear slope versus level-shift models (offset: log registered fleet; *n* = 8)

Model	k	Deviance	Pearson dispersion	QAICc	Quasi-Poisson estimates (95% CI)
Single log-linear slope	2	1,520.9	250.2	32.4	Slope 1.071 (1.051–1.091) per year
Step in 2020, slope thereafter	3	1,102.4	220.7	31.3	Step 1.042 (0.908–1.196); slope 1.081 (1.054–1.109)
Step in 2021, slope thereafter [1]	3	415.4	83.2	20.3	Step 1.219 (1.130–1.315); slope 1.061 (1.040–1.082)
Step in 2022, slope thereafter	3	2,321.5	476.9	50.8	Step 1.170 (0.988–1.386); slope 1.075 (1.008–1.146)
Segmented at 2021 (pre-slope, step, slope change)	4	250.1	62.5	27.0	Pre-2021 slope 0.955 (0.904–1.009); step 1.334 (1.174–1.517); post-2021 slope 1.061 (1.043–1.079)

[1] Preferred model (lowest QAICc; ΔQAICc = 12.1 versus the single slope). QAICc = −2·log L/ĉ + 2k + 2k(k+1)/(n−k−1), with ĉ = 62.5, the Pearson dispersion of the richest model. 'Step' models hold the fleet-adjusted injury rate flat before the step year and let it change log-linearly afterwards; the step is a one-off multiplicative change (a ratio, not a per-year rate). Estimates are quasi-Poisson (standard errors scaled by each model's own dispersion). With 40,000–130,000 injured riders per year, Poisson variation

is negligible, so the dispersion measures genuine year-to-year variation, which remains large (83) even in the preferred model; the single-slope estimates in Table S2a therefore describe the average annual change across a level shift. Year-on-year changes in the fleet-adjusted injury rate, 2019–2025: −5.2%, −3.7%, +32.2%, −0.6%, +5.4%, +11.5%, +4.7%.

Table S2c. Province-level models, 81 provinces (quasi-Poisson; rate ratios per +1 SD of the covariate). Outcome: fatality proportion (deaths per 100 reported casualties, log-casualties offset) unless noted

Model	Dispersion	Covariate (period average 2018–2024)	RR per +1 SD (95% CI)	p
Fatality proportion — main model	5.04	Hospital beds /100,000	1.097 (1.003–1.200)	0.042
		Physicians /100,000	0.921 (0.854–0.993)	0.033
		Dense-urban population share	0.850 (0.789–0.916)	<0.001
		log GDP per capita	0.947 (0.898–0.999)	0.047
Fatality proportion — + NUTS-1 region FE	2.62	Hospital beds /100,000	1.097 (1.025–1.175)	0.007
		Physicians /100,000	0.947 (0.889–1.008)	0.088
		Dense-urban population share	0.815 (0.755–0.879)	<0.001
		log GDP per capita	1.037 (0.988–1.089)	0.139
Fatality proportion — excl. İstanbul, Ankara, İzmir	3.52	Hospital beds /100,000	1.029 (0.940–1.126)	0.536
		Physicians /100,000	0.990 (0.905–1.083)	0.824
		Dense-urban population share	0.899 (0.845–0.956)	<0.001
		log GDP per capita	1.025 (0.969–1.084)	0.386
Post-admission share of deaths	—	Hospital beds /100,000	1.033 (0.971–1.098)	0.304
		Physicians /100,000	0.929 (0.881–0.981)	0.008
		Dense-urban population share	1.020 (0.967–1.076)	0.473
		log GDP per capita	1.032 (0.993–1.072)	0.110

Fatality-proportion models: deaths as outcome with log total casualties as offset. Post-admission model: deaths after referral as outcome with log total deaths as offset. Covariates standardized; all road users (province tables are not disaggregated by vehicle type).

Table S2d. Sensitivity of the exposure decomposition to the choice of baseline (predicted increase = baseline rate × (2025 fleet − baseline-year fleet); observed increase = 2025 deaths − baseline-year deaths)

Baseline	Rate (per 100,000 fleet)	Predicted increase to 2025 (baseline rate × fleet growth)	Observed increase (2025 − baseline year)	Predicted / observed (%)
2018	25.63	+999	+511	196
2019	20.77	+785	+642	122
2020	20.92	+753	+599	126
2021	20.75	+698	+557	125
2022	19.10	+567	+543	104
Mean 2019–2021	20.82	+787	+642	122
Mean 2019–2022	20.39	+770	+642	120

Across all baselines, fleet growth alone predicts 104–196% of the observed increase in annual rider deaths — i.e., at least the entire observed rise, with over-prediction under most baselines because the fleet-adjusted rate itself declined. The decomposition is an arithmetic restatement of the observed rate and fleet series, not an independent estimate.

Table S2e. Fatality-proportion and care-pathway models

Model	Family (dispersion)	OR/IRR per year (95% CI)	p
National fatality proportion (deaths ∣ casualties), 2018–2025	Quasi-binomial (3.10)	0.906 (0.891–0.922)	<0.001
Post-admission share of rider deaths, 2016–2025 (8 obs.)	Quasi-binomial (0.96)	1.020 (1.004–1.037)	0.016
Scene-death rate (per registered motorcycle), 2016–2025	Quasi-Poisson (2.77)	0.931 (0.910–0.953)	<0.001

Post-admission death rate (per registered motorcycle), 2016–2025	Quasi-Poisson (9.55)	0.952 (0.924–0.980)	0.001

Quasi-binomial standard errors scaled by √(max(1, Pearson dispersion)). The post-admission share is interpreted as a level rather than a trend: its upward drift is not robust to omitting 2025 (OR 1.014, 95% CI 0.991–1.038; Table S2g) and spans the change in the compiling source of the KGM series between the 2023 and 2025 editions (Table S3). Both component death rates decline, the scene rate faster. 2019 and 2024 are missing from the care-pathway series (editions not published).

Table S2f. Provincial model: univariable estimates, specification checks, and collinearity

Specification	Covariate	RR/OR per +1 SD (95% CI)	p	VIF
Univariable quasi-Poisson	Dense-urban share	0.799 (0.753–0.847)	<0.001	1.19
	Physicians /100,000	0.879 (0.827–0.934)	<0.001	2.19
	Hospital beds /100,000	1.027 (0.932–1.131)	0.59	1.98
	log GDP per capita	0.908 (0.850–0.969)	0.004	1.03
Quasi-binomial (deaths ∣ casualties), adjusted	Dense-urban share	0.848 (0.786–0.914)	<0.001	—
	Physicians /100,000	0.920 (0.852–0.993)	0.033	—
	Hospital beds /100,000	1.098 (1.003–1.203)	0.043	—
	log GDP per capita	0.947 (0.897–0.999)	0.047	—
Population denominator (deaths per pop. ≥15), adjusted	Dense-urban share	0.831 (0.740–0.932)	0.002	—
	Physicians /100,000	0.839 (0.745–0.945)	0.004	—
	Hospital beds /100,000	1.185 (1.035–1.358)	0.014	—
	log GDP per capita	0.912 (0.841–0.989)	0.025	—
Main model, NUTS-1 region-clustered SEs	Dense-urban share	0.850 (0.758–0.953)	0.005	—
	Physicians /100,000	0.921 (0.862–0.984)	0.015	—
	Hospital beds /100,000	1.097 (0.994–1.210)	0.065	—
	log GDP per capita	0.947 (0.858–1.046)	0.29	—

VIF: variance inflation factor of the z-scored covariates (all ≤ 2.2, indicating low collinearity). The quasi-binomial specification uses manually dispersion-scaled standard errors (Pearson dispersion 5.11). The population-denominator model estimates total mortality burden rather than severity conditional on a crash; the urbanization and physician-density associations persist in every specification.

Table S2g. Sensitivity of the headline trend estimates to the final years: full window, excluding 2025, and ending in 2023 (before TurkStat's İVME-based compilation of the 2024 and 2025 statistics)

Series (model); estimate per calendar year (95% CI)	Full window (to 2025)	Excluding 2025 (to 2024)	Ending in 2023
Rider deaths, 2019-anchored (primary)	0.983 (0.971–0.995)	0.986 (0.971–1.002)	0.984 (0.963–1.005)
Rider deaths, 2018-anchored	0.970 (0.953–0.986)	0.967 (0.945–0.990)	0.959 (0.929–0.989)
Non-motorcycle deaths (comparison series)	0.972 (0.939–1.007)	0.988 (0.948–1.030)	0.995 (0.934–1.060)
Ratio of trends, riders vs. comparison (pooled)	1.011 (0.951–1.075)	0.998 (0.926–1.075)	0.988 (0.880–1.110)
Injured riders, 2018-anchored	1.071 (1.051–1.091)	1.072 (1.044–1.100)	1.065 (1.025–1.107)
Injured riders, 2019-anchored	1.080 (1.058–1.103)	1.086 (1.054–1.118)	1.085 (1.034–1.138)
Fatality proportion (quasi-binomial OR), from 2018	0.906 (0.891–0.922)	0.904 (0.883–0.926)	0.902 (0.871–0.933)
Post-admission share of deaths (quasi-binomial OR), from 2016	1.020 (1.004–1.037)	1.014 (0.991–1.038)	1.014 (0.991–1.038)
Scene-death rate per registered motorcycle, from 2016	0.931 (0.910–0.953)	0.910 (0.893–0.928)	0.910 (0.893–0.928)
Post-admission death rate per registered motorcycle, from 2016	0.952 (0.924–0.980)	0.924 (0.905–0.942)	0.924 (0.905–0.942)

IRR or OR per calendar year (95% CI); Poisson scaling retained where the Pearson dispersion was ≤ 1, quasi-Poisson or quasi-binomial scaling otherwise, exactly as in Tables S2a and S2e. The care-pathway series has no 2024 value, so 'excluding 2025' and 'ending in 2023' coincide for those rows. Point estimates are stable across windows; intervals widen as observations are

removed, and the 2019-anchored mortality interval then includes unity (n = 6 and 5). The post-admission share trend is not robust to omitting 2025 and is interpreted as a level in the main text (Section 3.3). Motivation: the final year carries the greatest leverage in a short series, and TurkStat compiled the 2024 and 2025 statistics from the Ministry of Interior's İVME platform (metadata of the Road Traffic Accident Statistics); the 2025 figures are final (TurkStat does not revise these statistics).

Table S3. Care-pathway series (KGM): motorcycle rider deaths at the scene versus after referral to a health facility, 2016–2025, with reconciliation against the TurkStat death counts

Year	Compiling source (KGM edition)	At scene	After referral (≤30 days)	Total (KGM series)	TurkStat rider deaths	KGM vs. TurkStat (%)	After-care share (%)
2016	EGM/JGK	283	588	871	n.a.	—	67.5
2017	EGM/JGK	272	593	865	n.a.	—	68.6
2018	EGM/JGK	271	496	767	823	−6.8	64.7
2019	n.p.	n.p.	n.p.	n.p.	692	—	—
2020	EGM/JGK	219	466	685	735	−6.8	68.0
2021	EGM/JGK	229	458	687	777	−11.6	66.7
2022	EGM/JGK	209	487	696	791	−12.0	70.0
2023	EGM/JGK	267	607	874	1,014	−13.8	69.5
2024	n.p.	n.p.	n.p.	n.p.	1,228	—	—
2025	TurkStat	387	947	1,334	1,334	0.0	71.0

Sources: KGM annual traffic accident summaries — Table 8 of the 2016–2023 editions, compiled by KGM from police (EGM) and gendarmerie (JGK) crash data ('Kaynak: Emniyet Genel Müdürlüğü ve Jandarma Genel Komutanlığı trafik kaza verilerine göre düzenlenmiştir'), and Table 1.4 of the 2025 edition, which reproduces TurkStat's figures ('Kaynak: TÜİK'); TurkStat rider deaths from the vulnerable road user tables (Table 1 of the main text; the harmonized series begins in 2018). n.p.: edition not published online; n.a.: not available. Both sources count motorcycle drivers only, under the 30-day rule, yet the EGM/JGK compilations give 7–14% fewer deaths than TurkStat's final statistics for the same years, with the gap widening over time — consistent with TurkStat's validation of the police records and its integration, in cooperation with the Ministry of Health, of deaths within 30 days after referral to a health facility. The 2025 KGM figures reproduce TurkStat's and are therefore identical. The after-care share is computed within each edition, so numerator and denominator always come from the same source; because of the source break, the share is interpreted as a level and not as a trend (Section 3.3 of the main text). The EGM/JGK compilations also give 9–20% fewer involved motorcycles than TurkStat (42,533 vs. 46,545 in 2018; 39,597 vs. 45,753 in 2020; 54,037 vs. 64,479 in 2021; 58,452 vs. 71,270 in 2022; 74,120 vs. 92,565 in 2023), which is why Table 2 of the main text uses the TurkStat series throughout.

Table S4. Data source inventory

#	Variable(s)	Source and table	Years	Access
1	Rider deaths and injuries; e-scooter; totals; province, monthly, vehicle-type tables	TurkStat, Road Traffic Accident Statistics, 2025 edition — statistical tables (vulnerable road users t12; summary t1; vehicle type t7; provinces; months)	2010–2025	data.tuik.gov.tr (Aug 2026)
2	Registered motorcycle and total fleet (year-end)	TurkStat, Motor Vehicle Statistics — table t3 (vehicles by years)	2002–2025	data.tuik.gov.tr (Aug 2026)
3	At-scene vs post-referral rider deaths	KGM, Trafik Kazaları Özeti (annual PDF summaries) — Table 8 of the 2016–2023 editions (EGM/JGK compilation); Table 1.4 of the 2025 edition (TurkStat figures)	2016–2018, 2020–2023, 2025	kgm.gov.tr (Aug 2026)
4	Courier occupational accidents and fatalities (NACE H-53)	SGK Statistical Yearbooks, Section 3-1, Tables 3.1.1 and 3.1.2 (Art. 4-1/a insured)	2018–2025	sgk.gov.tr (Aug 2026)
5	Media-monitored courier deaths	Kurye Hakları Derneği annual reports (systematic media monitoring)	2022–2025 (partial)	kuryehaklari.org (Aug 2026)
6	112 stations; cases per station	Ministry of Health, Health Statistics Yearbook 2023 (Emergency Health Services chapter)	2002–2023	saglik.gov.tr (Aug 2026)
7	Transport-accident deaths, ICD-10 V01–V99 (validation)	TurkStat, Death and Causes of Death Statistics — causes by sex table	2022–2025	data.tuik.gov.tr (Aug 2026)

8	Province covariates (beds, physicians, urbanization, GDP p.c., etc.)	Compiled from TurkStat and Ministry of Health official series; period averages	2018–2024	Provided as Data S2 (DataS2_provinces.csv)
9	Province population aged ≥15 (denominator)	TurkStat, address-based population registration system	2025	data.tuik.gov.tr (Aug 2026)
10	Insured motorcycle fleet (compulsory MTPL policies); claim-victim series	Insurance Association of Türkiye (TSB), quarterly statistics, Q4 editions — 'Alt Branş Teminat Adet' (policy counts) and claim-victim tables, sub-branch 11 (motorcycles)	2018–2025	tsb.org.tr (Aug 2026)
11	Motorcycles involved in casualty crashes, by vehicle type	TurkStat, Road Traffic Accident Statistics — Institutional Quality Reports (Kurumsal Kalite Raporu), 2018–2025 editions, 'Kazaya karışan araç türü'	2018–2025	tuik.gov.tr/indir/kkr/ulastirma-haberlesme/TKIK (Sep 2026)

Table S5. Projected 2030 caseloads with 95% prediction intervals

Fleet scenario (2025→2030)	Fleet 2030 (million)	Outcome	Trend-based projection (95% PI)	Constant-2025-rate projection
Policy restraint (4%/yr)	8.65	Deaths	1,507 (1,355–1,676)	1,623
		Injured	237,218 (201,025–279,046)	158,868
Slowdown (8%/yr)	10.45	Deaths	1,820 (1,639–2,020)	1,960
		Injured	286,484 (243,061–335,270)	191,862
Recent trend (12%/yr)	12.53	Deaths	2,183 (1,965–2,419)	2,351
		Injured	343,615 (292,536–403,075)	230,123

Trend-based projections extrapolate the primary 2019–2025 quasi-/Poisson trend models to 2030; 95% prediction intervals from Monte Carlo simulation (20,000 draws; multivariate-normal parameter draws plus gamma–Poisson observation noise with the model dispersion; fixed seed in Code S1). Constant-rate projections apply the 2025 fleet-adjusted rates to the scenario fleets. All projections assume persistence of current trends without saturation and are planning scenarios, not forecasts.

Table S5-II. Sensitivity projection for injured riders in 2030: post-2021 slope of the level-shift model instead of the single 2019–2025 slope

Fleet scenario (2025→2030)	Single-slope trend projection (Table S5)	Post-2021-slope projection (95% PI)	Difference
Policy restraint (4%/yr)	237,218 (201,025–279,046)	212,802 (185,286–243,424)	−10.3%
Slowdown (8%/yr)	286,484 (243,061–335,270)	256,997 (224,587–294,550)	−10.3%
Recent trend (12%/yr)	343,615 (292,536–403,075)	308,248 (268,741–352,601)	−10.3%

The level-shift model (Table S2b-II, preferred by QAICc) implies a fleet-adjusted injury rate growing by 6.1% per year after 2021 (95% CI 4.0–8.2%) rather than the 8.0% per year of the single 2019–2025 slope; extrapolated to 2030 it lowers the injury caseload by about 10% under every fleet scenario. Prediction intervals by the same Monte Carlo procedure as Table S5 (20,000 draws; multivariate-normal parameter draws plus gamma–Poisson observation noise with the level-shift model's dispersion of 83.2; fixed seed in Code S1). The single-slope projections remain the primary planning scenarios in the main text; this table shows their sensitivity to the functional form of the injury trend.

Table S6. Correspondence of definitions across data sources

Concept	TurkStat traffic statistics	KGM annual summaries	SGK yearbooks	TSB insurance reports
Rider / casualty person	'Rider' = person driving the motorcycle (vulnerable road user tables); pillion passengers tabulated separately, not included; injured persons from the same tables	Driver deaths by vehicle type (pillion passengers not included); 2016–2023 editions compiled from EGM/JGK data, 2025 edition reproduces TurkStat (Table S3)	Insured employee involved in a registered occupational accident (any role)	Claim victims (any person indemnified)
Death	Road-crash death within 30 days (international definition, since 2015)	Deaths at scene vs. within 30 days after referral to a health facility (driver	Fatality from a registered occupational accident (Act No. 5510)	Fatality claim victims, tabulated by payment year (not event year)

		deaths by vehicle type); the EGM/JGK-compiled editions count 7–14% fewer deaths than TurkStat's final statistics		
Injury / accident	Police- and gendarmerie-recorded injured persons; motorcycles involved in casualty crashes (institutional quality reports, 2018–2025)	Involved vehicles in casualty crashes — death or injury — (one crash may involve several vehicles); 9–20% below the TurkStat counts in the EGM/JGK-compiled editions	Reported occupational accidents among insured employees	Disability and treatment claim victims (payment year)
Motorcycle	Casualty tables: 'motosiklet' drivers, with moped (motorlu bisiklet) drivers tabulated separately (2025: 90 deaths, 2,523 injured); registration statistics: all two- and three-wheeled motor vehicles incl. ATVs and registered mopeds (no separate moped category)	Vehicle type of the involved vehicle (motosiklet)	Not vehicle-based: economic activity NACE H-53 (postal and courier activities)	MTPL sub-branch 11, 'Motosiklet ve Yük Motosikleti'
Exposure denominator	Registered year-end fleet (Motor Vehicle Statistics)	—	Insured employees under Art. 4-1/a in H-53	Policies in force at year-end (insured fleet)
Coverage caveats	National; casualty capture may improve with EMS reach	Full-country editions only; 2019 and 2024 unpublished; source break between the 2023 and 2025 editions	Formal employees only; contractors/informal riders excluded	Insured vehicles only (65–71% of registrations, stable)

The TSB-insured share of the registered fleet (65–71% in every study year, drifting by about 1% per year) bounds the effect of the denominator choice on the trend estimates at about 1% per year (Section 3.2 of the main text). Claim-victim series are recorded by payment year and lag the event year; they were therefore not used as outcome series.

Figure S1. Provincial fatality proportions with 95% confidence intervals, 2025

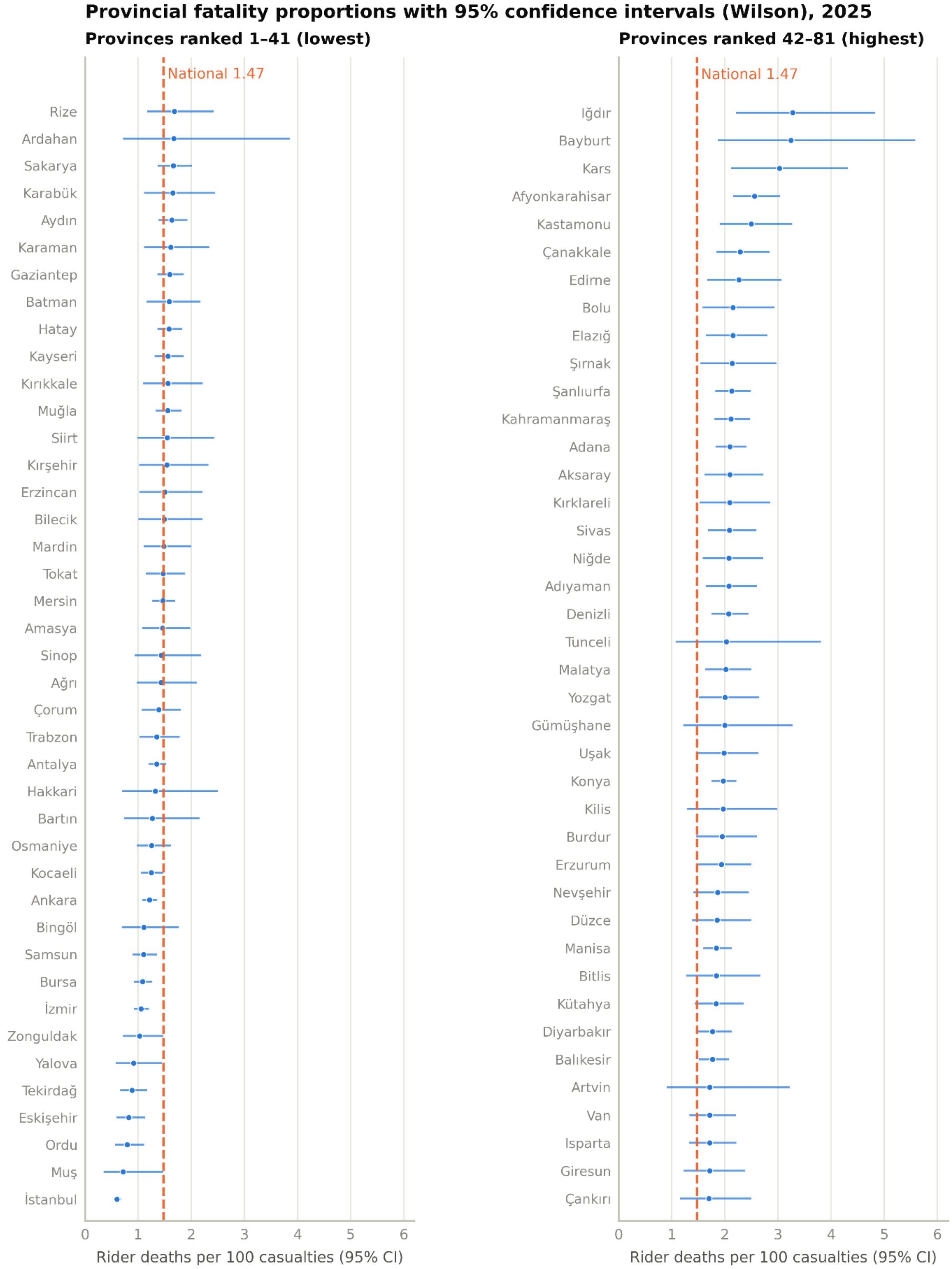

The all-road-crash fatality proportion (deaths per 100 reported casualties) for each of the 81 provinces in 2025, with exact (Wilson) 95% confidence intervals, shown in two panels for legibility (left: provinces ranked 1–41, lowest; right: ranked 42–81, highest; within each panel ordered from the lowest value at the bottom to the highest at the top). The dashed line marks the national value (1.47). The extensively overlapping intervals caution against reading the provincial ranking as a league table.

Code S1 and Data S1–S4

Code S1 (CodeS1_analysis.ipynb; also provided as CodeS1_analysis.py) is a self-contained analysis script that reproduces all rates, trend models (including the insured-fleet sensitivity and the pooled interaction test), the exposure decomposition and its baseline sensitivity, the fatality-proportion and care-pathway models, the province-level models with all specification checks, the AICc comparison for mortality and the QAICc comparison of the injury level-shift models (Table S2b-II), the leave-2025-out and 2018–2023 sensitivity estimates (Table S2g), the Monte Carlo 2030 projections and their post-2021-slope sensitivity (Tables S5 and S5-II), and the insured-share arithmetic, from the accompanying data files; CodeS1_figures.py redraws Figures 1–5 of the main text and Figure S1 from the same files. The data files are: Data S1 (DataS1_national_series.csv — national casualty, fleet, and comparison series), Data S2 (DataS2_provinces.csv — province-level outcomes and covariates), Data S3 (DataS3_crash_involvement.csv — TurkStat motorcycles involved in casualty crashes, 2018–2025, with the EGM/JGK compilation for comparison), and Data S4 (DataS4_insured_series.csv — TSB insured-fleet, KGM care-pathway, and SGK courier-denominator series). Running Code S1 on Data S1–S4 reproduces every estimate, table, and figure reported in the article.
